# C-Jun N-terminal kinase controls TDP-43 accumulation in stress granules induced by oxidative stress

**DOI:** 10.1186/1750-1326-6-57

**Published:** 2011-08-08

**Authors:** Jodi Meyerowitz, Sarah J Parker, Laura J Vella, Dominic CH Ng, Katherine A Price, Jeffrey R Liddell, Aphrodite Caragounis, Qiao-Xin Li, Colin L Masters, Takashi Nonaka, Masato Hasegawa, Marie A Bogoyevitch, Katja M Kanninen, Peter J Crouch, Anthony R White

**Affiliations:** 1 Department of Pathology, The University of Melbourne, Victoria, 3010, Australia; 2 Ludwig Institute for Cancer Research, Austin Hospital, Harold Stokes Building, 145-163 Studley Road, Heidelberg, Victoria, 3084, Australia; 3 Bio21 Molecular Science and Biotechnology Institute, The University of Melbourne, Parkville, Victoria, 3052, Australia; 4 Department of Biochemistry and Molecular Biology, The University of Melbourne, Parkville, Victoria, 3052, Australia; 5 The Mental Health Research Institute, Parkville, Victoria, 3052, Australia; 6 Department of Molecular Neurobiology, Tokyo Institute of Psychiatry, Tokyo 156-8585, Japan

**Keywords:** TDP-43, stress granules, JNK, kinases, oxidative stress, paraquat, hnRNP

## Abstract

**Background:**

TDP-43 proteinopathies are characterized by loss of nuclear TDP-43 expression and formation of C-terminal TDP-43 fragmentation and accumulation in the cytoplasm. Recent studies have shown that TDP-43 can accumulate in RNA stress granules (SGs) in response to cell stresses and this could be associated with subsequent formation of TDP-43 ubiquinated protein aggregates. However, the initial mechanisms controlling endogenous TDP-43 accumulation in SGs during chronic disease are not understood. In this study we investigated the mechanism of TDP-43 processing and accumulation in SGs in SH-SY5Y neuronal-like cells exposed to chronic oxidative stress. Cell cultures were treated overnight with the mitochondrial inhibitor paraquat and examined for TDP-43 and SG processing.

**Results:**

We found that mild stress induced by paraquat led to formation of TDP-43 and HuR-positive SGs, a proportion of which were ubiquitinated. The co-localization of TDP-43 with SGs could be fully prevented by inhibition of c-Jun N-terminal kinase (JNK). JNK inhibition did not prevent formation of HuR-positive SGs and did not prevent diffuse TDP-43 accumulation in the cytosol. In contrast, ERK or p38 inhibition prevented formation of both TDP-43 and HuR-positive SGs. JNK inhibition also inhibited TDP-43 SG localization in cells acutely treated with sodium arsenite and reduced the number of aggregates per cell in cultures transfected with C-terminal TDP-43 162-414 and 219-414 constructs.

**Conclusions:**

Our studies are the first to demonstrate a critical role for kinase control of TDP-43 accumulation in SGs and may have important implications for development of treatments for FTD and ALS, targeting cell signal pathway control of TDP-43 aggregation.

## Background

Amyotrophic lateral sclerosis (ALS) is a fatal adult-onset neurodegenerative disease in which the function of motor neurons in the spinal cord and brain progressively deteriorates. ALS is by far the most prevalent form of motor neuron disease. Patients with ALS rarely survive more than 3-5 years after diagnosis with respiratory failure the most common cause of death [1]. Approximately 5% of patients with ALS have a positive family history of the disorder. The first pathological mutations identified in ALS were in superoxide dismutase 1 (SOD1) and account for around 20% of familial ALS cases [2]. That discovery has been the basis for most ALS research in the past decade, and animal models containing SOD1 mutant transgenes have provided important insights into SOD1-mediated neurotoxic effects. However, SOD1 mutations only account for 1-2% of all ALS cases [3].

Frontotemporal dementia (FTD) is the second most common cause of presenile dementia, affecting people in their 50s and 60s [4,5]. There are several clinical phenotypes and the historical neuropathological classification included either frontotemporal lobar degeneration with tau positive (FTLD-tau) or ubiquitin-positive (FTLD-U) inclusions [4,5]. The observation that some ALS patients developed cognitive deficits with frontal lobe degeneration resembling FTLD-U has led to the belief that ALS and FTD with FTLD-U might involve a clinical spectrum of neurodegenerative illnesses [5].

In 2006, TAR DNA binding protein 43 (TDP-43) was identified as the major protein constituent of ubiquitinated neuronal inclusions in FTLD-U and in non-SOD1 ALS cases [6,7]. This led to the re-classification of FTLD-U to FTLD-TDP-43, and TDP-43-positive ALS and FTLD-TDP-43 cases are now referred to collectively as primary TDP-43 proteinopathies [8]. These findings also provided further support for the concept of FTD and ALS as diseases within the same broad clinical spectrum. Subsequently, TDP-43-positive inclusions have been identified in a number of neurodegenerative diseases. In these cases, the TDP-43 identification is referred to as a secondary TDP-43 proteinopathy [8]. While the role of abnormal TDP-43 accumulation in both primary and secondary TDP-43 proteinopathies is not yet fully understood, the identification of TDP-43 mutations associated with ALS and FTD (~40 at present) has provided clear evidence that altered TDP-43 processing can be a primary cause of neurodegeneration and is not just a secondary phenomenon [9,10].

TDP-43 is a 414 amino acid protein of the heterogeneous nuclear ribonucleoprotein (hnRNP) family and consists of two RNA recognition motifs and a C-terminal glycine rich region [8,11]. It has a number of reported roles including transcription, pre-mRNA splicing, and transport and stabilization of mRNA [8]. The protein is normally localized to the nucleus and has a classical bipartite nuclear localization sequence [12]. TDP-43 contains two caspase 3 consensus cleavage sites leading to formation of C-terminal fragments (CTFs) of 35 kDa and 25 kDa that are excluded from the nucleus [8]. The majority of TDP-43 mutations occur in the C-terminal region and CTFs are commonly identified in ALS and FTD inclusions.

In post-mortem tissue from ALS and FTD, the hallmark neuropathological features include loss of TDP-43 expression in the nucleus together with accumulation of TDP-43 in cytoplasmic inclusions. These inclusions are enriched in ubiquitinated and hyperphosphorylated (phospho-Ser409/410) TDP-43 and there can be substantial enrichment of CTF-TDP-43 [8,11]. Recent cell studies have shown that transfection with CTF-TDP-43 can accurately re-capitulate the histopathological findings of ALS and FTD with accumulation of cytosolic ubiquitinated and phosphorylated CTF-TDP-43 aggregates [13-15]. In addition, transfection with these constructs can result in neurotoxicity and cell death although the pathways involved are not known [14].

However, while these studies have recapitulated findings of post-mortem disease tissue, they have told us little of the early disease processes associated with abnormal TDP-43 metabolism, particularly in sporadic TDP-43 proteinopathies which account for > 90% of ALS (and FTD) cases. A new insight into TDP-43 accumulation is developing through studies identifying TDP-43 association with RNA stress granule proteins [16,17]. Stress granules (SGs) are cytoplasmic sites of stalled mRNA pre-initiation complexes induced by oxidative changes, heat shock or osmotic stress where the cell stalls mRNA translation of non-critical proteins to shift energy expenditure to key repair and survival proteins [18]. Recent studies have shown that under stress, TDP-43 is recruited to SGs in a variety of cells [16,17,19,20]. Initially Moisse et. al. [21] reported that TDP-43 localized to SGs after axotomy in mice. Subsequently, studies in cells revealed that acute cell stress induced TDP-43 SG association and this was dependent on residues 216-315 and the first RNA recognition motif [19]. While the same group reported a lack of TDP-43 association with SG markers in ALS tissues, subsequent work by Volkening et al. [22] reported an association between TDP-43 and staufen in ALS spinal cord tissue. TDP-43 SG co-localization in ALS and FTLD-U has since been reported by Liu-Yesucevitz et al., [17] and FUS, another hnRNP protein associated with ALS, has also been identified in ALS SGs [23,24]. Liu-Yesucevitz et al. [17] also reported that TDP-43 may associate with SGs through interaction with SG proteins such as TIA-1 and this has been supported by studies on TDP-43 association with a number of SG proteins [20,25]

However, while these studies have advanced our understanding of the early stages of TDP-43 aggregation, the majority of this research has been performed in cells exposed to acute and highly toxic treatment with sodium arsenite, the standard means of inducing SGs [17,19,20]. In addition, much of our knowledge has been gained through generation of CTF-TDP-43 over-expression in transfected cells. There is a lack of understanding about the processes involved in endogenous TDP-43 aggregation during chronic oxidative stress. As the majority of ALS and FTD cases involve no known mutation in TDP-43 and the slow disease process characteristic of neurodegeneration involves chronic oxidative and nitrosative stresses [2,26], it is critical to determine how these factors affect TDP-43 SG cytosolic accumulation. Moreover, SG proteins have a high propensity to aggregate and over-expression of highly aggregating CTF fragments may not accurately re-capitulate the underlying mechanistic processes involved in endogenous TDP-43 aggregation and association with SGs during chronic stress. Therefore, we investigated the effects of mild, chronic oxidative and nitrosative stress on endogenous TDP-43 in neuronal-like cell cultures. Our findings revealed that in contrast to acute stress, chronic oxidative stress induced several features consistent with TDP-43 proteinopathies including loss of nuclear TDP-43, accumulation of diffuse TDP-43 in the cytosol, formation of a 35 kDa C-terminal fragment and accumulation of TDP-43 in SGs, some of which revealed ubiquitination. Importantly, our findings revealed that TDP-43 localization to SGs was controlled by c-Jun N-terminal kinase (JNK). Inhibition of JNK also modulated TDP-43 accumulation in SGs induced by sodium arsenite and in cells transfected with CTF-TDP-43 constructs. Our data also indicated that the aggregation of TDP-43 may be associated with JNK modulation of hnRNP-TDP-43 interactions and SG localization.

## Results

To investigate the effects of chronic stress on TDP-43 metabolism, we first determined optimal concentrations of oxidative and nitrosative stress inducers in SH-SY5Y neuronal-like cultures. Cells were treated overnight with each compound at a range of concentrations and the cell viability was determined by MTT assay and cell death was measured using an LDH assay (not shown). Additional File 1 shows the selected concentrations used for further investigation. The concentrations shown in Additional File 1 induced mild but significant reductions in cell viability overnight. However, except for 2 mM paraquat (24 ± 3.2% cell death) and 75 μM rotenone (32 ± 4.6% cell death), no change to LDH release was observed compared to untreated controls. These doses were used to mimic sub-lethal chronic stress conditions relevant to brain or spinal cord neurons during disease *in vivo*.

### Nitrosative stress inducers mediate altered TDP-43 processing

**Figure 1:**
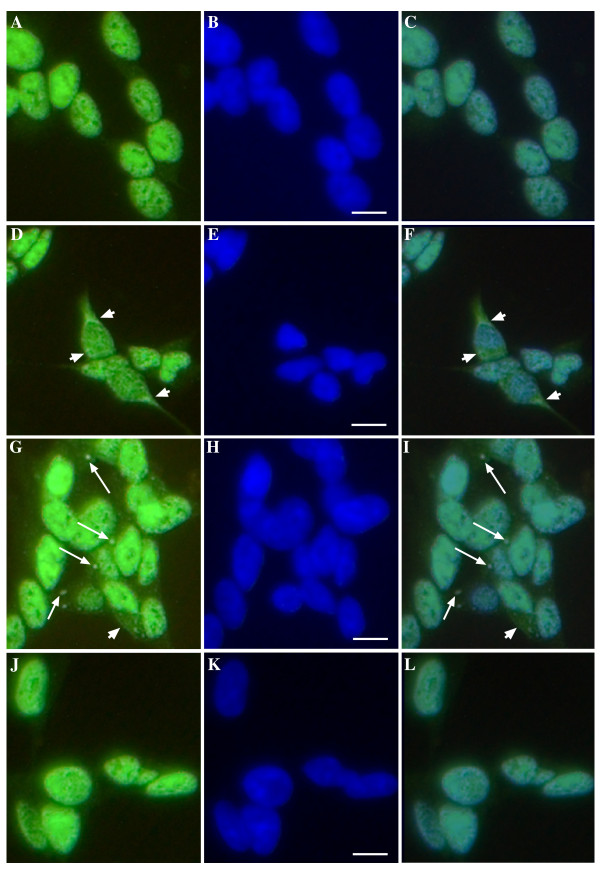
**Effect of SIN-1, paraquat and arginine on TDP-43 localization in SH-SY5Y cells**. Cells were exposed overnight with 0.1 mM SIN-1, 1 mM paraquat or 1 mM arginine and TDP-43 localization was examined by immunofluorescence. **A-C**: untreated, **D-F**: SIN-1, **G-I**: paraquat, **J-L**: arginine. Green = TDP-43, blue = DAPI. Right-hand panel = merged images of TDP-43 and DAPI. Arrowheads show diffuse cytosolic TDP-43. Arrows show aggregated cytosolic TDP-43. Bar = 10 μm. Representative images from three separate experiments performed in duplicate or triplicate.

Treatment of SH-SY5Y cells with inducers of nitrosative stress resulted in changes to sub-cellular distribution of TDP-43. Compared to untreated controls (Figure 1A-C), SIN-1, a peroxynitrite donor caused a frequent, evenly distributed, diffuse accumulation of TDP-43 in the cytosol of treated cells (Figure 1D-F). In contrast, paraquat, an inhibitor of the mitochondrial electron transport chain and inducer of superoxide/peroxynitrite stress (a common feature in neurodegeneration), induced substantial and varied cytoplasmic accumulation of TDP-43 including aggregates of TDP-43 resembling RNA SGs (Figure 1G-I). Arginine (nitric oxide precursor) had no consistent effect (Figure 1J-L).

### Paraquat induces a robust cell model of TDP-43 proteinopathy

**Figure 2:**
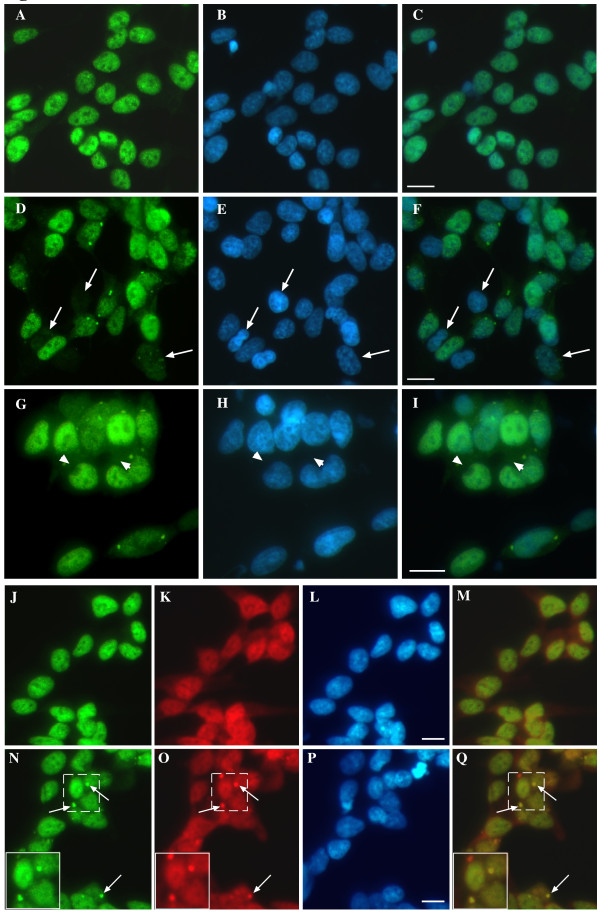
**Induction of cytosolic TDP-43 accumulation and SGs by paraquat in SH-SY5Y cells**. Cells were exposed overnight to 1 mM paraquat and TDP-43 localization was examined by immunofluorescence. **A-C**: untreated, **D-I**: paraquat treated. Arrows show loss of nuclear TDP-43. Arrowheads show diffuse cytosolic TDP-43. Green = TDP-43, blue = DAPI. Right-hand panels = merged images. **J-Q: **Cells were exposed to 1 mM paraquat overnight and TDP-43 and HuR localization was measured by immunofluorescence. **J-M**: untreated, **N-Q**: paraquat treated. Green = TDP-43, Red = HuR, Blue = DAPI. **M **and **Q **are merged images from TDP-43 and HuR panels. Arrows indicate stress granules. Inset shows higher magnification of TDP-43 and HuR positive SGs. Bar = 10 μm. Representative images from four separate experiments performed in duplicate or triplicate.

Further examination of TDP-43 in paraquat-treated cells revealed multiple features reported for human TDP-43 proteinopathies. Paraquat-treated cells frequently showed clear loss of nuclear TDP-43 (Figure 2D-F), accumulation of diffuse TDP-43 in the cytosol (Figure 2G-I) and formation of cytoplasmic aggregates. Interestingly, these changes were not always observed in the same cells suggesting that loss of nuclear TDP-43 expression and accumulation in the cytosol may been caused by different stress-mediated processes. To determine if the cytosolic aggregates of TDP-43 induced by paraquat were SGs, cells were co-stained for the SG marker, HuR. The majority of TDP-43 aggregates co-localized with HuR although there were also additional HuR-positive SGs that lacked TDP-43 (Figure 2N-Q). Quantitative analysis revealed that 66 ± 8% of paraquat-induced SGs that were positive for HuR were also positive for TDP-43 and that SG formation correlated to increasing toxicity of paraquat (Additional File 2). TDP-43 also frequently co-localized with the SG marker, TIA-1 (data not shown). We examined the time course of TDP-43 SG formation and found that TDP-43 only accumulated into SGs between 8 and 20 hr after exposure to paraquat. This is in contrast to the rapid accumulation of TDP-43 into SGs reported for arsenite or osmotic stress [19,20]. Our findings were also observed in retinoic-acid differentiated SY5Y neuronal-like cells, confirming that these changes can occur in non-dividing SY5Y cells (Additional File 2H-K).

**Figure 3:**
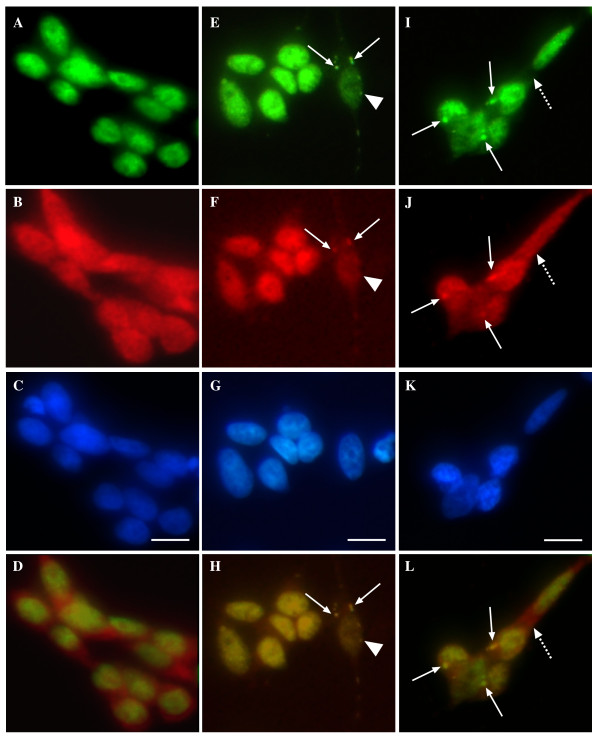
**Treatment of SH-SY5Y neurons induces co-localization of TDP-43 and ubiquitin in SGs**. Cells were treated overnight with 1 mM paraquat and localization of TDP-43 and ubiquitin was determined by immunofluorescence. **A-D**: untreated, **E-L**: paraquat treated. Green = TDP-43, red = ubiquitin, blue = DAPI. **D**, **H **and **L **represent merged images of panels above. Solid arrows indicate co-localization of TDP-43 and ubiquitin in SGs. Arrowhead indicates lack of co-localization of TDP-43 and ubiquitin in one SG in panel **F**. Dotted arrow indicates lack of co-localization of diffuse TDP-43 and ubiquitin. Bar = 10 μm. Representative images from three separate experiments performed in duplicate or triplicate.

We extended the investigation of this model further by examining if TDP-43-positive SGs revealed presence of the protein aggregate marker ubiquitin, also a hallmark feature of the ubiquitinated inclusions in ALS and FTLD-U in FTD. Interestingly, our study revealed that a number of the TDP-43-positive SGs co-localized with ubiquitin (Figure 3F and 3J). 24 ± 6% of TDP-43-positive SGs were also positive for ubiquitin indicating that only a portion of the SGs may progress to ubiquitinated protein aggregates (Figure 3). Diffuse TDP-43 did not consistently co-localize with ubiquitin (Figure 3J). Whether the ubiquitination of the SGs was associated directly with the TDP-43 or ubiquitination of alternative SG proteins is uncertain. Due to the relatively low numbers of cells containing ubiquitinated SGs and lack of a method for purifying SGs, it was not possible to determine if the ubiquitinated protein in the SGs was specifically TDP-43.

Interestingly, we did not observe phosphorylated TDP-43 associated with the SGs (Additional File 3A-F). This was confirmed by Western blot analysis that detected no increase in phosphorylated TDP-43 or phosphorylated CTF-TDP-43 post-exposure to paraquat (Additional File 3G). It is possible that more prolonged treatment of cells is required to induce phosphorylation or that the correct cellular kinases are not present or not-localized to SGs. Alternatively the TDP-43 may be phosphorylated on sites different to the 409/410 site. However, the combination of clear nuclear loss of TDP-43, diffuse cytosolic accumulation, aggregation and ubiquitination under mild stress provided a unique model for investigating the early processes in abnormal TDP-43 processing associated with ALS and FTD.

### Paraquat induces formation of caspase-dependent and caspase-independent TDP-43 SGs

**Figure 4:**
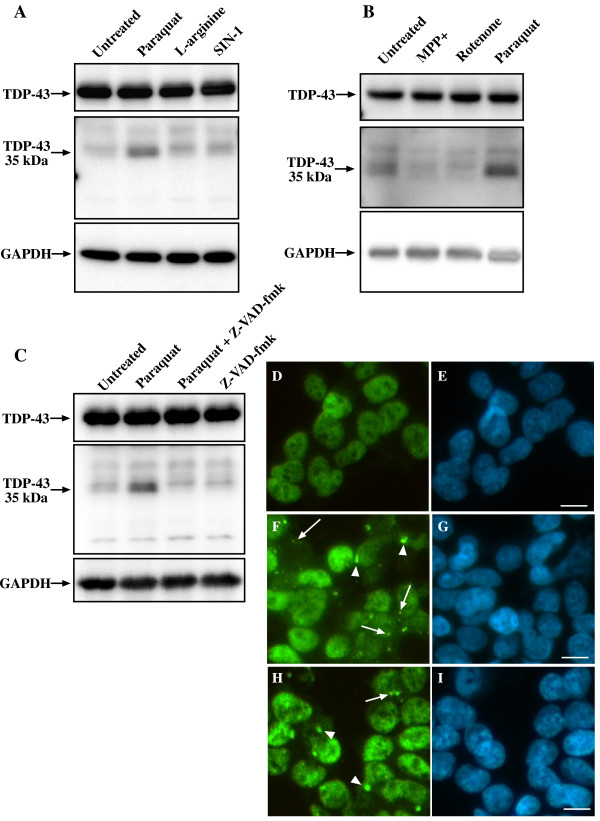
**Paraquat treatment induces up-regulation of a caspase-dependent TDP-43 fragment**. SH-SY5Y cells were treated overnight with 1 mM paraquat, 1 mM arginine, 100 μM SIN-1 (**A**), or 1 mM paraquat, 2 mM MPP+, 0.075 mM rotenone (**B**) and TDP-43 expression was determined by immunoblot. **C**: Cells were treated overnight with 1 mM paraquat with and without 50 μM Z-VAD-fmk (caspase inhibitor) and TDP-43 expression was determined by immunoblot. Middle panels represent a longer exposure to visualize the 35 kDa band. **D-I: **Cells were treated overnight with paraquat in the absence (**F-G**) and presence (**H-I**) of Z-VAD-fmk and examined for TDP-43-positive SGs. Green = TDP-43, Blue = DAPI. Arrows indicate TDP-43-positive SGs ≤ μm. Arrowheads indicate TDP-43-positive SGs ≥ 1 μm. Bar = 10 μm. Representative images from two-three separate experiments.

One of the hallmark neuropathological features of TDP-43 proteinopathies is the formation of C-terminal TDP-43 fragments (CTF-TDP-43), often of 35 or 25 kDa in mass [6,8]. Cell studies have re-capitulated features of end-stage TDP-43 proteinopathies through expression of these fragments which aggregate and co-localize with SG proteins [13,27]. Therefore, we examined if our paraquat model also induced CTF-TDP-43. The SH-SY5Y cells revealed basal expression of a 35 kDa TDP-43 band even in untreated cultures. This is consistent with previous observations [28]. Western blot analysis of paraquat-treated cells revealed the increased expression of this 35 kDa CTF-TDP-43 (Figure 4A). Interestingly, none of the additional mitochondrial inhibitors or nitrosative stress inducers tested significantly elevated 35 kDa CTF-TDP-43 (Figure 4A and 4B). This was despite inducing a similar loss of cell viability (Additional File 1). These findings suggested that formation of TDP-43 SGs may be specifically associated with CTF-TDP-43 as previously supported by studies involving transfection of cells with TDP-43 CTF constructs [16]. Co-treatment of cells with paraquat and the broad-spectrum caspase inhibitor, Z-VAD-fmk, resulted in a complete inhibition of increased 35 kDa CTF-TDP-43 expression (Figure 4C). This supported previous studies demonstrating that 35 kDa TDP-43 CTFs are generated by caspase-cleavage at a DETD consensus site within the NLS of TDP-43 [29,30]. However, we found that while inhibiting CTF-TDP-43 generation with Z-VAD-fmk partially inhibited TDP-43 SG formation (Figure 4H), the effect was not complete. Treatment of cultures with Z-VAD-fmk reduced the number of cells containing TDP-43-positive SGs from 18 ± 8% to 8 ± 2% (P < 0.05). This inhibitory effect was mainly due to a reduction in cells containing smaller TDP-43-positive SGs as there was no loss of large (≥ 1 μm) TDP-43 SGs in Z-VAD-fmk treated cells, despite a complete inhibition of enhanced 35 kDa CTF formation. In our cultures, no change was observed to a faint 25 kDa CTF-TDP-43 (Figure 4C), ruling out involvement of this fragment in TDP-43 SG formation. Nishimoto et al. have also reported that the 25 kDa form is not involved in TDP-43 SG formation [30]. These findings strongly suggest that while paraquat treatment enhanced 35 kDa CTF-TDP-43 formation, this was not sufficient for TDP-43 SG formation.

### Induction of cytosolic TDP-43 accumulation by paraquat is not mediated through mitochondrial inhibition

**Figure 5:**
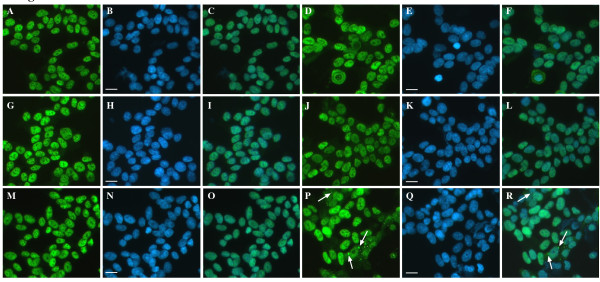
**Treatment of SH-SY5Y cells with different mitochondrial inhibitors did not induce TDP-43 aggregation**. Cells were treated with vehicle control (**A-C**), 0.075 mM rotenone (**D-F**), 1 mM 3-NP (**G-I**), 2 mM MPP+ (**J-L**), 5 mM sodium azide (**M-O**) or 1 mM paraquat (**P-R**). Cells were analyzed for TDP-43 localization by immunofluorescence. Green = TDP-43, blue = DAPI. C, F, I, L, O and R represent merged images of adjacent TDP-43 and DAPI panels to the left. Arrows indicate TDP-43 SGs in paraquat-treated cells. Bar = 10 μm. Representative images from three separate experiments performed in duplicate or triplicate.

As paraquat is a mitochondrial electron transport chain inhibitor, we compared paraquat treatment with alternative inhibitors of cellular respiration to determine if mitochondrial impairment induced TDP-43 SGs. Figure 5P-R shows that only paraquat induced cytosolic accumulation and formation of TDP-43-positive SGs after an overnight treatment. Other mitochondrial inhibitors including rotenone (Figure 5D-F), 3-NP (Figure 5G-I), MPP+ (Figure 5J-L) and sodium azide (Figure 5M-O) had no effect on TDP-43 despite being applied at concentrations that induced the same or increased level of mild cell toxicity (Additional File 1). We then determined if the alternative mitochondrial respiration inhibitors induced HuR-positive SGs that lacked TDP-43. However, as shown in Additional File 4, no HuR-positive SGs were observed in cells after overnight treatment with the mitochondrial inhibitors. These observations showed that the ability of paraquat to induce loss of nuclear TDP-43, cytosolic accumulation and SGs is not solely attributable to its ability to inhibit mitochondrial activity per se. These findings suggest that the effects of paraquat on TDP-43 are more likely associated with specific pathways of oxidative or nitrosative stress induction that differ from the other mitochondrial inhibitors.

### JNK controls TDP-43 localization to SGs during oxidative stress

**Figure 6:**
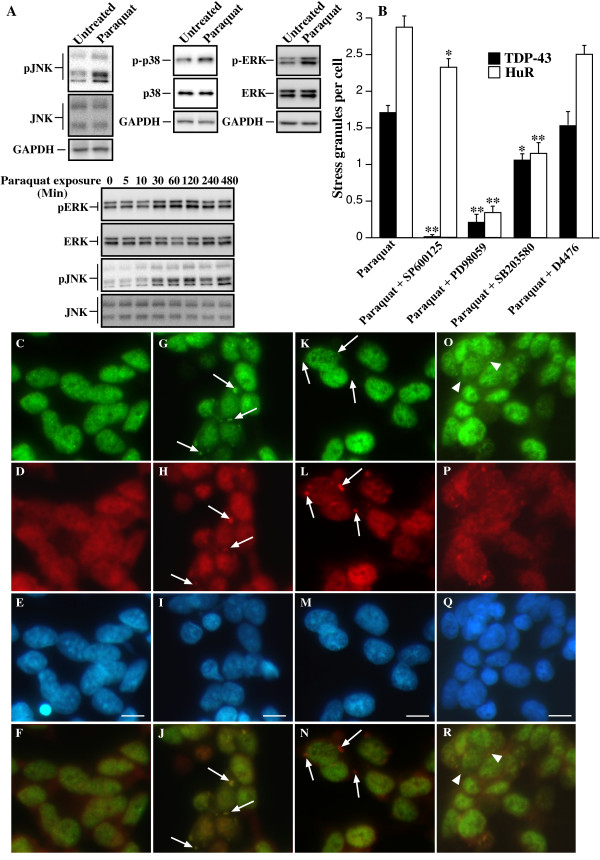
**Treatment of SH-SY5Y cells with paraquat induces JNK-dependent accumulation of TDP-43 into SGs**. Cells were treated with 1 mM paraquat overnight. Where indicated, cells were co-treated with 10 μM SP600125 (JNK inhibitor), PD98059 (ERK inhibitor), SB203580 (p38 inhibitor) or D4476 (CK1 inhibitor). Cells were examined for phosphorylation of kinases by immunoblot and accumulation of TDP-43 and HuR by immunofluorescence. **A**: Cells were treated with paraquat and examined after overnight incubation for activation of JNK, p38 and ERK. In addition, cells were incubated with paraquat and examined at different time points from 0-480 min (8 hr) for ERK and JNK activation. Lower panels for each image indicate that total kinase expression is unchanged, upper panels indicate changes to phorphorylated forms. **B**: Cells were treated with paraquat in the presence or absence of kinase inhibitors and the number of TDP-43 and HuR SGs was determined. *p < 0.05, **p < 0.01. n = minimum of 500 cells counted across multiple coverslips and separate experiments for each inhibitor. **C-F**: Untreated, **G-J**: Paraquat treated, **K-N**: Paraquat + SP600125 showing loss of TDP-43 but not HuR SGs, **O-R**: Paraquat + SP600125 showing loss of TDP-43 SGs but not diffuse cytosolic TDP-43. Green = TDP-43, red = HuR, blue = DAPI. Bottom panels indicate merged images of TDP-43 and HuR panels above. Arrows indicate SGs, arrowheads indicate diffuse TDP-43. Bar = 10 μm. Representative images from two-four separate experiments performed in duplicate or triplicate.

It has been reported previously that kinases can control cytoplasmic localization and SG accumulation of hnRNPs such as hnRNP A1 and hnRNP K. This includes p38, JNK and ERK-dependent modulation of hnRNP sub-cellular accumulation [31-36]. Of these, JNK has been clearly established as a critical stress-activated kinase [37] and is central to toxic effects of paraquat [38,39]. Therefore, we examined if modulation of JNK activity affected TDP-43-positive SG formation. Initially, we determined if paraquat induced activation of JNK and p38 as previously reported [38]. After overnight treatment with 1 mM paraquat robust activation of JNK and ERK was observed with weaker p38 activity (Figure 6A). A time course of activation revealed elevated JNK and ERK phosphorylation after 30-60 min with maximal activation at 2 hr (Figure 6A). No early activation of p38 was observed (data not shown). Subsequent co-treatment of cultures with paraquat and the JNK inhibitor, SP600125, resulted in almost complete inhibition of TDP-43-positive SGs, with little effect on the presence of HuR-positive SGs (Figure 6B and 6C-R). This was paralleled by inhibition of JNK phosphorylation (Additional File 4K). The numbers of SGs per cell was used as a more consistent indicator than total number of cells containing SGs. However, in paraquat-treated cultures, the number of cells containing one or more TDP-43-positive SGs was 18 ± 8% of all cells. Co-treatment with SP600125 and paraquat reduced this to 0.22 ± 0.06% of cells (P < 0.01). No cells containing SGs were observed in control cultures.

As SP600125 is not entirely specific for JNK, we also tested the effect of BI-78D3, a specific JNK inhibitor on TDP-43 SG formation [40] and found that this induced the same effect as SP600125 (data not shown). This was further supported by the fact that inhibition of another SP600125 target kinase, casein kinase 1 (CK1) with a CK1 inhibitor (D4476), had no effect on TDP-43 or HuR SG formation (Figure 6B). Additional confirmation of the specific role for JNK in TDP-43 accumulation in stress granules was obtained through JNK knockdown. Treatment with combined siRNA against JNK1 and JNK2 significantly reduced JNK expression (Additional File 5A). Subsequent treatment with paraquat resulted in almost no TDP-43-positive stress granules while still inducing HuR-positive stress granules (Additional File 5B-M).

**Figure 7:**
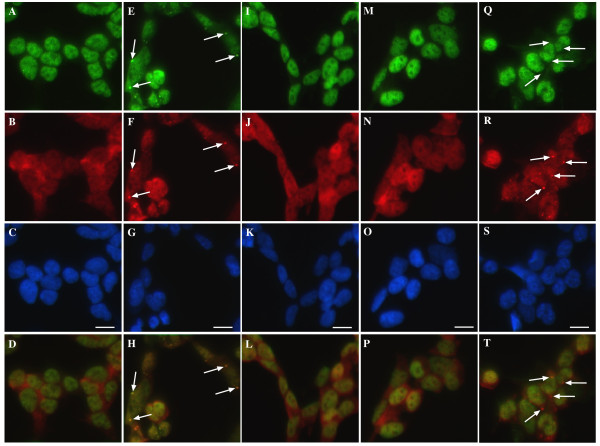
**Treatment of SH-SY5Y cells with paraquat induces ERK and p-38-dependent accumulation of TDP-43 and HuR-positive SGs**. Cells were treated overnight with 1 mM paraquat in the absence or presence of 10 μM PD98059 (ERK inhibitor), SB203580 (p38 inhibitor) or SP600125 (JNK inhibitor) and immunofluorescence analysis of TDP-43 and HuR was performed. **A-D: **Untreated control, **E-H: **paraquat-treated, **I-L: **paraquat and PD98059, **M-P: **paraquat and SB203580, **Q-T: **paraquat and SB600125. Green = TDP-43, red = HuR, blue = DAPI. Arrows indicate SGs. Bottom panel indicates merged images from TDP-43 and HuR panels above. Bar = 10 μm. Representative images from three separate experiments performed in duplicate or triplicate.

In contrast, inhibition of ERK with PD98059 had a substantial inhibitory effect on both TDP-43 and HuR-positive SG formation (Figure 6B and Figure 7I-L compared to A-D). In paraquat-treated cultures, 27.4 ± 7% of cells contained HuR-positive SGs (no HuR-positive SGs were observed in control cultures). This was reduced to 1.5 ± 0.3% after treatment with PD98059 (P < 0.01). A parallel decrease in the number of cells containing TDP-43-positive SGs was observed (reduced from 18 ± 8% to 0.99 ± 0.2% of cells, P < 0.01). These effects were also confirmed using the additional ERK inhibitor, U0126 and Raf inhibitor, GW5074 (data not shown). A somewhat weaker effect was observed on TDP-43 and HuR SG formation by SB203580, an inhibitor of p38 (Figure 6B and Figure 7M-P compared to A-D). SB203580 reduced the number of cells containing HuR-positive SGs from 27.4 ± 7% to 12.9 ± 1.7% (P < 0.01) and cells containing TDP-43-positive SGs from 18 ± 8 to 7.2 ± 2.1% (P < 0.01). Although ERK was activated earlier than p38 by paraquat, the inhibition of HuR and TDP-43-positive SGs by inhibitors of both kinases is consistent with our observations that SGs were not detected until after 8 hr of paraquat exposure (data not shown). This suggests that different kinases may have a role in SG formation over the prolonged exposure to paraquat with JNK controlling TDP-43 association and ERK and p38 affecting TDP-43 and additional SG protein accumulation. This is the first report of JNK and additional kinases controlling TDP-43 localization to SGs. The fact that inhibition of JNK resulted in almost complete abrogation of TDP-43-positive SGs with little effect on HuR localization to SGs indicated that JNK is potentially a key controller of TDP-43 (and possibly other hnRNP) association with SGs rather than simply mediating SG formation *per se*.

As the majority of studies on SGs involve acute (0.5 - 1 hr) treatment with toxic doses of stress inducers such as arsenite, heat shock or osmotic stress, we examined whether a short-term treatment with paraquat induced JNK-controlled TDP-43 SG formation. Interestingly treatment of cells for 1 hr with up to 5 mM paraquat had no effect on HuR or TDP-43 (data not shown), demonstrating that paraquat-mediated SG formation is a longer term process requiring prolonged incubation for TDP-43 to localize to SGs. The data are more consistent with a role for paraquat in prolonged oxidative stress than impairment of mitochondrial function and suggest that paraquat or other chronic inducers of TDP-43 SG formation may provide useful models to mimic the slow progression of disease-associated changes in ALS or FTD.

### JNK controls TDP-43 SG association in different cell-types

To determine if the effect of JNK inhibition on TDP-43 localization with SGs in SH-SY5Y cells was specific for this cell-type, we compared this to additional cell-lines treated with paraquat. Treatment of HeLa cells and U87MG glial cells overnight with 1 mM paraquat resulted in TDP-43-positive SGs (Additional File 6). Extensive numbers of TDP-43 SGs were observed in HeLa cells (~28% of cells) while SG positive cells in U87MG cultures were rare (~2% of cells) (Additional File 6). No paraquat-induced SGs were observed in HEK293 or human fibroblasts (GSM2069) (not shown). Co-treatment of HeLa cultures with paraquat and SP600125, dramatically reduced formation of TDP-43-postive SGs, with only a limited effect on the presence of HuR-positive SGs analogous to SP600125 treatment of SH-SY5Y cells (data not shown). These findings demonstrate that paraquat induces TDP-43-positive SGs in different cell-types and JNK-mediated control of TDP-43 with SGs is not specific for one cell line but appears to be a consistent feature of chronic stress-induced SG formation.

### JNK partially controls TDP-43 association with SGs in arsenite stress

**Figure 8:**
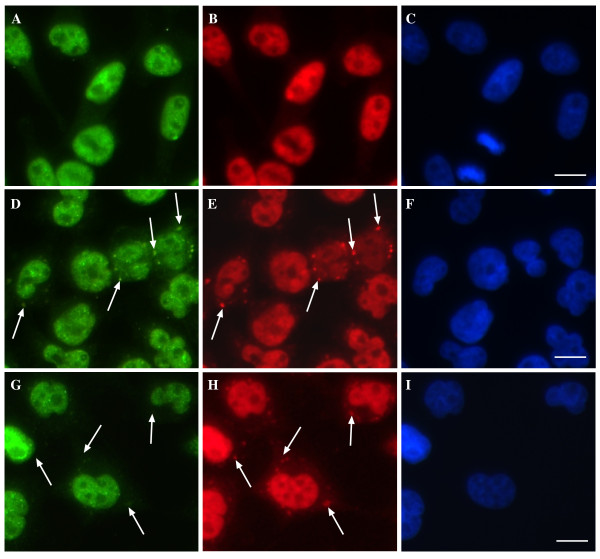
**Treatment of HeLa cells with sodium arsenite induces JNK-dependent TDP-43 SGs**. HeLa cells were treated for 1 hr with 0.5 mM sodium arsenite and cells were examined for TDP-43 and HuR localization by immunofluorescence. **A-C**: untreated, **D-F**: sodium arsenite-treated, **G-I**: sodium arsenite and SP600125. Green = TDP-43, red = HuR, blue = DAPI. Arrows indicate SGs. Bar = 10 μm. Representative images from three separate experiments performed in duplicate or triplicate.

We examined whether inhibition of JNK also affected localization of TDP-43 in cells exposed to sodium arsenite, the most commonly used SG inducer. Sodium arsenite is also known to induce JNK activation [41]. Initially, we compared the effect of sodium arsenite on TDP-43 in SH-SY5Y cells at a concentration that induced the same level of toxicity as paraquat did overnight. 50 μM sodium arsenite overnight induced 58% cell viability (compared to 57% cell viability in cells treated with 2 mM paraquat overnight, Additional File 1B). However, this level of toxicity with sodium arsenite did not cause cytosolic TDP-43 SGs. We also examined short term treatment of cells with sodium arsenite (0.5 mM sodium arsenite for 1 hr) and while this induced robust HuR SGs, few cells revealed TDP-43 co-localization. Given the lack of TDP-43 SGs in the sodium arsenite-treated SH-SY5Y cells, we treated HeLa cells with sodium arsenite (0.5 mM 1 hr). This treatment induced widespread TDP-43 and HuR-positive SGs (Figure 8D-F). To determine if JNK activity was responsible for TDP-43 accumulation in sodium arsenite-treated cells, cultures were co-treated with SP600125 and paraquat. Interestingly, we observed a 46% decrease in the number of cells positive for TDP-43 SGs (Figure 8G-I). As with inhibition of paraquat-treated SH-SY5Y cells, SP600125 had little effect on HuR SGs (Figure 8G-I). This partial, but significant, inhibition of TDP-43 localization to SGs by JNK inhibition suggests that JNK has a role in TDP-43-SG interaction during acute sodium arsenite treatment but other factors (e.g. other kinases) are also involved.

### JNK inhibition partially modulates aggregation of transfected CTF-TDP-43

**Figure 9:**
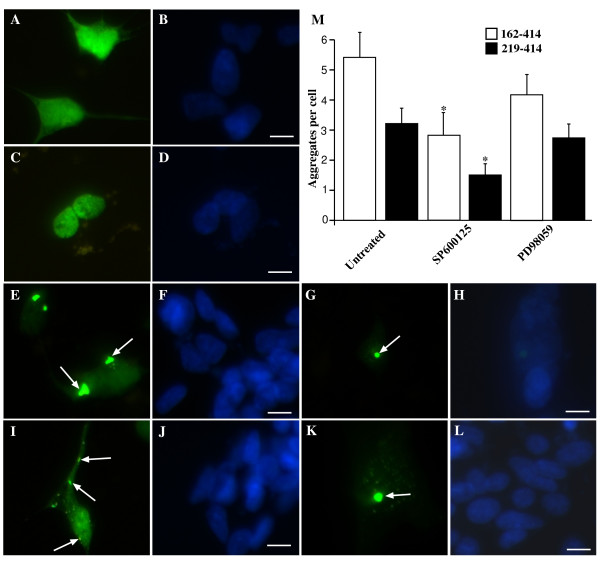
**Aggregation of CTF-TDP-43 162-414 and 219-414 in SY5Y cells is partially associated with JNK**. SH-SY5Y cells were transfected with GFP-tagged constructs for vector control (**A-B**), full length TDP-43 (**C-D**), CTF-TDP-43 219-414 (**E-H**) or CTF-TDP-43 162-414 (**I-L**). Green = TDP-43-GFP, blue indicates DAPI. Arrows indicate TDP-43 aggregates. Bar = 10 μm. Cells were transfected with CTF-TDP-43 162-414 or 219-414 for 24 hr and co-treated with SP600125 (JNK inhibitor) (**G-H **and **K-L**) or PD98059 (ERK inhibitor) (not shown) for a further 24 hr. The number of TDP-43-GFP aggregates per cell was determined (**M**). **p < 0.01. Representative images from two separate experiments performed in triplicate.

Next we investigated whether JNK controls aggregation of transfected CTF-TDP-43 constructs. SH-SY5Y cells were transfected with GFP-tagged vector control, full length TDP-43, CTF-TDP-43 162-414 or CTF-TDP-43 219-414 for 48 hrs. As expected, no aggregates of TDP-43 were observed in cells exposed to vector control (Figure 9A-B) or full length TDP-43 (Figure 9C-D). In contrast, CTF-TDP-43 162-414 or 219-414 induced cytoplasmic aggregates in cells after 48 hr consistent with previous reports [15] (Figure 9E-F and 9I-J). We then treated cultures with SP600125 after 24 hr (to allow transfection to stabilize) and examined the formation of TDP-43 aggregates after a further 24 hr incubation. While treatment with SP600125 did not reduce the number of cells containing aggregated TDP-43, there was a significant decrease in the number of aggregates per cell in cultures transfected with TDP-43 162-414 (Figure 9K-L and 9M) and 219-414 (Figure 9G-H and 9M). ERK inhibition induced a small decrease in number of aggregates but this was not significant. These findings suggested that the aggregation of these CTF-TDP-43 fragments maybe partially affected by JNK. This could be due to a role for basal JNK activity in modulating CTF-TDP-43 aggregation or alternatively, early aggregation of the CTF-TDP-43 fragments could induce cell stress that promotes further CTF-TDP-43 aggregation via JNK activation. This stress may then accelerate aggregation in some cells.

### JNK inhibition of TDP-43 SG formation is not due to inhibition of 35 kDa CTF-TDP43 expression

**Figure 10:**
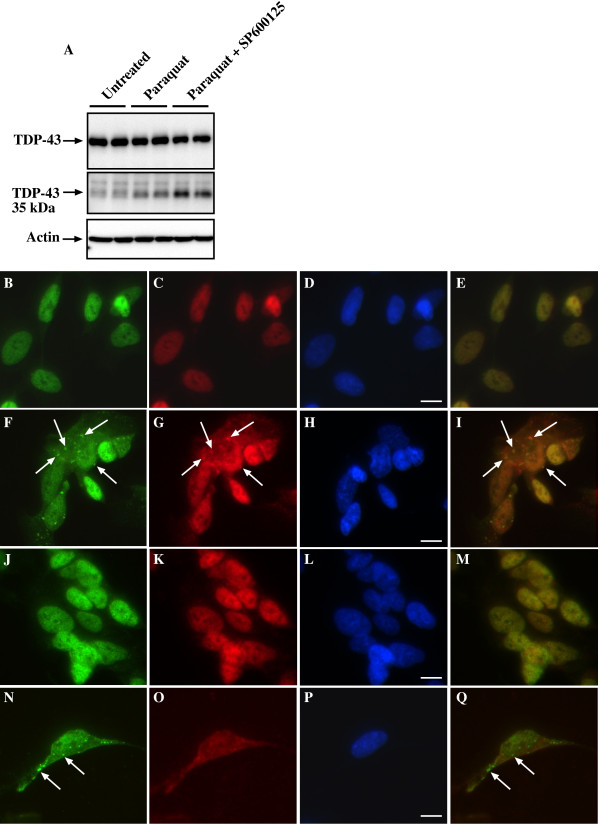
**JNK inhibition blocks association of hnRNP K and TDP-43 with SGs**. SH-SY5Y cells were treated with 1 mM paraquat overnight in the presence and absence of SP600125. **A**: Cells were immunoblotted for full length TDP-43 and 35 kDa CTF-TDP-43, **B-Q**: Cells were treated with paraquat and SP600125 and examined for TDP-43, hnRNP K or hnRNP A1 by immunofluorescence. **B-E**: untreated, labeled for TDP-43 (green) and hnRNP K (red); **F-I**: paraquat-treated, labeled for TDP-43 (green) and hnRNP K (red); **J-M**: paraquat and SP600125, labeled for TDP-43 (green) and hnRNP K (red); **N-Q **paraquat-treated, labeled for TDP-43 (green) and hnRNP A1 (red). Right-hand panel indicates merged images from TDP-43 and hnRNP panels. Arrows indicate SGs. Bar = 10 μm. Representative images from three separate experiments performed in duplicate or triplicate.

Interestingly, while JNK inhibition blocked TDP-43 incorporation in SGs, it did not have a substantial effect on inhibiting accumulation of diffuse cytosolic TDP-43 or prevent loss of nuclear TDP-43 induced by paraquat treatment (Figure 6C-R). This finding provided further support for the role of JNK in modulating cytosolic TDP-43 incorporation into SGs rather than affecting upstream processes leading to loss of nuclear TDP-43 and accumulation of TDP-43 in the cytosol. Additional support for this was obtained when we examined the effect of JNK inhibition on 35 kDa CTF-TDP-43 accumulation. As shown in Figure 10, co-treatment of cultures with the JNK inhibitor, actually led to an increase in detectable levels of the 35 kDa CTF-TDP-43 rather than inhibit its formation. This is consistent with the data in Figure 4 demonstrating that the formation of TDP-43-positive SGs was not fully prevented by inhibiting 35 kDa CTF-TDP-43 formation using a caspase inhibitor.

### JNK inhibition blocks association of hnRNP K and TDP-43 with SGs

In order to obtain an insight into the potential mechanism by which JNK controls TDP-43 association with SGs during chronic stress, we examined co-localization with other hnRNPs. Previous studies have reported that TDP-43 binds to hnRNPs including hnRNP A1 and K [25,42] and that stress kinases including JNK can control the cellular localization and SG association of these hnRNPs [31-36]. Analysis of TDP-43 and hnRNP A1 during paraquat stress did not reveal any co-localization within SGs (Figure 10N-Q). In contrast, paraquat-treated cells revealed significant co-localization of hnRNP K and TDP-43 in SGs (Figure 10F-I). Interestingly, JNK inhibition fully blocked both TDP-43 and hnRNP K SG accumulation (Figure 10J-M). As hnRNP K is known to bind to TDP-43, associate with SGs and is phosphorylated by JNK, these findings suggest that modulation of TDP-43 SG association by JNK could be controlled through binding to hnRNP K. However, a comprehensive analysis of hnRNP interactions with JNK and TDP-43 is required to determine if this is the mechanism occurring in paraquat-treated cells and other stress-associated conditions leading to TDP-43 accumulation.

## Discussion

Despite considerable research into TDP-43 in the past five years, little is known about the earliest pathological events associated with TDP-43 accumulation in ALS and FTD. In this study, we have developed a model of oxidative stress to investigate changes to endogenous TDP-43 processing during cell stresses that reflect the chronic nature of ALS and FTD. We show here that mild stress induced by paraquat, a well-characterized mitochondrial inhibitor and oxidative stress inducer, induced changes to TDP-43 metabolism that closely re-capitulated features observed in brain and/or spinal cord of FTD and ALS patients. These changes included clearance of TDP-43 from cell nuclei, accumulation of diffuse TDP-43 in cytosol, aggregation into SGs, ubiquitination of a portion of these SGs and increased expression of the 35 kDa CTF-TDP-43. These are all considered important hallmarks of TDP-43 proteinopathies [6,8]. Importantly, we also found these changes to TDP-43 metabolism in differentiated neurons and additional cell-lines demonstrating that this was not a cell-specific effect. In addition, short term treatment of cells with paraquat (1 hr) had no effect on TDP-43, providing strong support for chronic cell stress as an important mediator of TDP-43 abnormal processing as observed in ALS and FTD CNS tissues.

The key finding of this study was that cell kinase activity and in particular, JNK activation, modulates TDP-43 localization to SGs. This is the first report of TDP-43 localization controlled by kinase activity. This process is perhaps not surprising as previous reports describe the nuclear-cytoplasmic movement and SG localization of alternative hnRNPs and HuR. Habelhah et. al., have shown that phosphorylation of hnRNP K by ERK can modulate cytoplasmic accumulation [34]. In a separate study they also demonstrated that hnRNP K is phosphorylated by JNK at serine 216 and serine 353 [43]. Moreover, p38 phosphorylates hnRNP A1 inducing SG localization [35,36]. There is also evidence that JNK modulates localization and activity of HuR [44]. Importantly, several studies have shown that HuR and hnRNP A1 and K as well as other hnRNPs directly bind TDP-43 [25,42,45]. Interestingly this is mediated through interaction at the C-terminal region of both proteins. The C-terminal domain of TDP-43 is where the majority of known ALS/FTD disease mutations have been identified [11]. Moreover, there are key JNK phosphorylation consensus sites (Ser/Thr-Pro) within the C-terminal region of hnRNP K and HuR [43]. It is possible that kinase (especially JNK) phosphorylation of hnRNPs modulates interaction with TDP-43, thus mediating SG association. Alternatively, specific phosphorylation of hnRNPs may simply target them to SGs and due to TDP-43 association with these hnRNPs, it becomes localized to SGs where hnRNPs are present. Further support for an hnRNP-TDP-43 association was found in our model where we showed that JNK inhibition blocked localization of both TDP-43 and hnRNP K to SGs. This is particularly interesting as hnRNP K is phosphorylated by JNK [43] and the phosphorylation site lies within the hnRNP C-terminal domain that interacts with TDP-43 in studies on other hnRNPs [42]. Further support for this was shown by the fact that there was no specific localization of hnRNP A1 with paraquat-induced TDP-43 SGs in our study. Interestingly, the only JNK phosphorylation consensus site on hnRNP A1 is in the N-terminal region (Ser7/Pro8) rather than in the C-terminal region that would interact with TDP-43. In addition to these findings, we observed that JNK inhibition did not decrease CTF-TDP-43 generated by paraquat treatment and in fact increased expression. This indicated that JNK is more likely to be controlling localization of cytoplasmic TDP-43 to SGs similar to that reported for other kinases and hnRNPs, rather than modulating the formation of CTF-TDP-43. Whether it is CTF-TDP-43 or full length TDP-43 or both that is aggregating into SGs in this model remains to be seen. Due to the loss of nuclear TDP-43 expression and the fact that CTF-TDP-43 only accounted for approximately 10% of total TDP-43 on Western blots, strongly suggested that the SGs probably contained full length TDP-43 or a mixture of full length and CTF-TDP-43.

There must also be additional factors associated with TDP-43 localization to SGs. JNK activation is not specific for paraquat and in fact, alternative mitochondrial inhibitors used in this study also induce JNK activation [38]. Phosphorylation of JNK is a common downstream effect of oxidative and other cells stresses. The specificity of paraquat to induce JNK-mediated localization of TDP-43 may be related to specific sub-cellular localization of activated JNK or modulation of additional co-factors. Considerable investigation will be required to delineate the specific processes induced by paraquat that leads to JNK-mediated TDP-43 SG accumulation and how these may relate to neurodegenerative diseases such as ALS.

**Figure 11:**
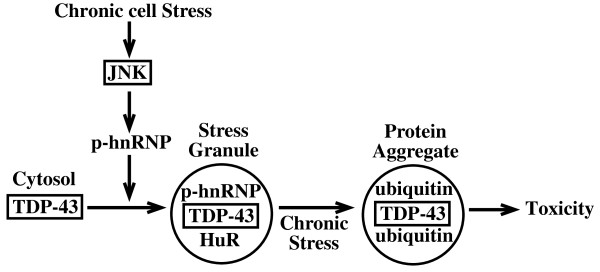
**Schematic of proposed stress-mediated TDP-43 accumulation into SGs**. Chronic cell stress (eg oxidative stress) induces activation of stress kinases such as JNK. This may phosphorylate hnRNPs such as hnRNP K resulting in phosphoryated forms (p-hnRNP). This could modulate interaction with TDP-43 and association with SGs. Prolonged stress may lead to progression of SGs to protein aggregates containing TDP-43 and subsequent cell toxicity.

While some recent studies have reported a possible association between TDP-43 and TIA-1 [16,17], these have been demonstrated with transfected cells and no clear evidence of endogenous TDP-43-TIA-1 interaction was identified. Moreover, TIA-1 does not contain JNK consensus sites and there are no reports of JNK control of TIA-1 localization. We believe that the data presented here are more consistent with a potential interaction between TDP-43 and hnRNP K (Figure 11). However, further studies will be required to demonstrate specific interaction in this chronic stress model and to determine if mutation of the C-terminal JNK phosphorylation site on hnRNP K prevents TDP-43 association with SGs. It was also clear from our findings that additional kinases can control TDP-43 and probably a range of hnRNPs during stress. It will take a considerable effort to delineate the role of p38, ERK and additional kinases on TDP-43 accumulation both *in vitro *and *in vivo*.

We also observed partial JNK-mediated control of TDP-43 localization to SGs induced by sodium arsenite, the most common method used for SG induction. The lack of complete inhibition of TDP-43 SG accumulation was possibly related to the fact that sodium arsenite rapidly induces SGs (minutes), while paraquat had no effect on TDP-43 in short-term treatment even at very high doses. This suggests that while sodium arsenite and paraquat induce SGs and both involve JNK, there are different cellular mechanisms involved in short term and longer term SG formation. This is consistent with the previously reported concept that different stresses have diverse affects on SG formation [18]. In this context, we feel that our paraquat-based mild oxidative stress model is an important tool for delineating TDP-43 SG association as it occurs under mild stress conditions expected in chronic neurodegenerative diseases and better re-capitulates the features of TDP-43 proteinopatheis than sodium arsenite. It is possible that the latter, (ie acute sodium arsenite exposure) rapidly drives SG formation in cells that are experiencing high levels of toxicity. As shown in Additional File 1B, treatment with 500 μM sodium arsenite overnight results in almost total loss of cell viability as compared to only 15% decrease in viability with 1 mM paraquat overnight.

Whether JNK directly modulates TDP-43 is not known. TDP-43 does not contain known consensus sites for JNK, p38 or ERK. However, it does contain two putative JNK binding domains (RxxxKxxxLxV and KxxRxxxxVxF) at 98-108 and 224-235 respectively. It remains a possibility that JNK binds to TDP-43 and acts as a scaffolding protein affecting SG localization. While no other studies have demonstrated a TDP-43-JNK association, a previous report described a role for a JNK-interacting protein, WDR62 in SG formation [41]. Interestingly, they reported that inhibition of JNK during sodium arsenite treatment increased the number of SGs (in HEK293 cells) but decreased the size of the granules. This is in contrast to our finding in HeLa cells where we found a partial decrease in TDP-43 SG association but no observable changes to HuR SG formation with SP600125. In addition, JNK inhibition did not block SG formation by paraquat as determined by HuR staining but did block TDP-43 and hnRNP K localization. However, these differences are again likely to be due to acute sodium arsenite treatment compared to longer paraquat treatment used here, different cell lines and different markers of SGs eg HuR and TIA-1. Importantly, the findings show that different model systems may give a range of different outcomes and in terms of understanding TDP-43 pathological changes, it will be important to ensure that the model gives an accurate reflection of the disease processes. With that in mind, we are currently investigating TDP-43 metabolism in primary neuronal and glia cell cultures as this may be a more accurate model system to understand TDP-43 SG dynamics.

The role for stress kinases such as JNK and p38 in ALS has been suggested through recent studies. SOD1 ALS models have shown enhanced activity of these kinases as well as modulation of ERK [46-50]. Interestingly, a recent report by Ayala et al. [51] found ERK aggregates in stressed cells and ALS tissues and inhibition of ERK lead to increased TDP-43 aggregation in cultures. While these affects appear to contrast with our own findings, the differences may reflect different intensity and form of stress as well as different cell models and time frame. It will be important to determine the kinetics of ERK and other kinases activation across the disease course in ALS. A single report on JNK activation in ALS patients has described increased activity in astrocytes but not neurons in spinal cord of these patients [52]. We found that paraquat induced TDP-43 aggregation in both neuronal-like and astroglial cell lines in this study. Whether JNK or additional kinases are associated with early changes to TDP-43 accumulation in vivo is not known due to the difficulty of obtaining relevant early disease tissues. It is likely that with the current development of multiple animal models of TDP-43 proteinopathy that re-capitulate human disease neuropathology, we will be able to determine the early events in TDP-43 processing. It is also uncertain what role hnRNPs have in determining TDP-43 aggregation in ALS or FTD. While a large number of hnRNPs have been shown to bind to TDP-43 and many are associated with SGs, their role in ALS and FTD has not been established. It is important to note, however, that several recent studies have shown that TDP-43 and FUS are associated with SG marker proteins in ALS tissues [17,24].

An important outcome from this study is that kinases may be an important target for therapeutic intervention in ALS and FTD. Should further studies show that kinase activation controls TDP-43 aggregation especially early in disease, it may be possible to inhibit this process with kinase inhibitors. Interestingly, the only approved treatment for slowing ALS disease progression, Riluzole, is known to modulate stress kinase activity [53], and kinase modulators have been discussed previously as possible therapeutic agents for ALS.

## Conclusions

In summary, it has been difficult to accurately model endogenous aberrant TDP-43 in cell models. Treatment of cells with sodium arsenite or osmotic stress induces robust TDP-43 containing SGs however, these models have not recapitulated the broad features of TDP-43 mis-metabolism observed in ALS and FTD brain and spinal cord tissues in a manner consistent with transfection of CTF-TDP-43 constructs. The latter however, are likely to be prone to spontaneous aggregation when over-expressed and may not represent an accurate model of the cellular control of TDP-43 processing during chronic stress. Likewise, although studies with mutant TDP-43 constructs can help to understand the disease processes, the majority of ALS and FTD cases are sporadic and probably involve only endogenous, non-mutated TDP-43. Our model has recapitulated a number of features of aberrant endogenous TDP-43 metabolism including loss of nuclear staining, accumulation of diffuse cytoplasmic TDP-43, formation of CTF-TDP-43, aggregation into SGs and ubiqitination of a portion of these SGs indicating the possible transition to irreversible protein aggregates. The aggregation of TDP-43 into SGs is controlled by JNK and SG formation is controlled by additional kinases and these factors are associated with chronic stress. Future studies will be required to fully delineate the mechanism by which kinases control TDP-43 aggregation and whether this is involved in TDP-43 aggregation in vivo. These findings may have important implications for identifying potential therapeutic targets for intervention in ALS and FTD.

## Methods

### Materials

4',6' Diamino-2-phenylindole dihydrochloride (DAPI) was obtained from Invitrogen (Mount Waverley, Victoria, Australia). (3-(4,5-Dimethylthiazol-2-yl)-2,5-diphenyltetrazolium bromide (MTT), N, N'-dimethyl-4,4'-bipyridinium dichloride (paraquat), rotenone, 1-methyl-4-phenylpyridinium (MPP+), sodium azide, sodium arsenite, 3-nitropropionic acid (3-NP) and 3-Morpholinosyndnomine (SIN-1) were from Sigma Aldrich (Sydney, NSW, Australia) and LDH assay kit was purchased from Roche Diagnostics (Castle Hill, NSW, Australia). SP600125, PD98095, SB203580 were purchased from Merck Biosciences (Melbourne, Victoria, Australia). BI-78D3 and D4476 were purchased from Tocris Bioscience (Ellisville, Melbourne, Victoria, Australia). Z-VAD-fmk was obtained from Promega (Sydney, Australia).

Polyclonal TDP-43 antisera were purchased from Proteintech Group (Chicago, IL, USA). Monoclonal antisera to the phosphorylated form of TDP-43 (ser409/410) were obtained from Cosmo Bio (Tokyo, Japan). Antisera to ubiquitin were from Santa Cruz Biotechnology (Santa Cruz, CA, USA). Monoclonal antisera to hnRNP A1 and hnRNP K were purchased from Abcam (Waterloo, Australia). Monoclonal antisera to HuR were obtained from Invitrogen (Mount Waverley, Victoria, Australia). Antisera to total and phosphorylated forms of p38, ERK and JNK, as well as antibodies to actin and GAPDH were purchased from Cell Signalling Technologies (Arundel, Queensland) or BD Bioscience (North Ryde NSW, Australia).

## Methods

### Cell Culture

The cell lines used in this study were human neuroblastoma SH-(SY5Y) cell line, human epithelial HeLa cell line, human embryonic kidney cell line (HEK293), human fibroblast cell line (GSM2069) and human astroglial U87MG cell line. Cells were passaged and maintained in DMEM plus 5% FBS (HeLa and HEK293 cells), DMEM/F12 plus 10% FBS (SH-SY5Y and U87MG cells) or BME plus 10% FCS (GSM2069 fibroblasts). To induce differentiation, SY5Y cells were treated with 10 μM retinoic acid for 7 days. Differentiation was confirmed by morphological changes (neurite extension) and up-regulated expression of synaptophysin, tyrosine hydroxylase and VMAT2. All cells were grown in 5% CO_2 _at 37°C.

### Cell viability and cell lysis assays

Assays for cell viability (MTT) and cell lysis (LDH) were performed as previously described [28].

### Exposure of cell to stress

Undifferentiated cells were grown in 24 or 6-well plates or on 12 mm coverslips (for immunofluorescence) for 2-3 days before experiments (~80% confluent). Differentiated SH-SY5Y cells were cultured in the presence of retinoic acid for 7 days before experiments. Inducers of nitrosative stess (arginine, paraquat and SIN-1) or oxidative stress (rotenone, 3-NP, sodium azide, MPP+, sodium arsenite and paraquat) were prepared in dH_2_O and added at indicated concentrations and the medium was briefly mixed by aspiration. Incubations were performed for periods stated in individual experiments. Where indicated, cells were co-treated with kinase inhibitors (SP600125 (JNK), BI-78D3 (JNK), PD98095 & U0126 (ERK), SB203580 & SB202190 (p38), D4476 (casein kinase 1) from stock solutions prepared at 10 mM in DMSO. Control cultures were treated with vehicle alone. For immunoblotting, cells were harvested into Phosphosafe Extraction Buffer (Merck Biosciences, San Diego, CA, USA) containing protease inhibitor cocktail (Roche Diagnostics) and stored at -80°C until use. For immunofluorescence studies, cells were grown on glass coverslips and fixed by treating with 4% paraformaldehyde for 30 min.

### siRNA knockdown of JNK

ON-TARGETPlus human JNK1 siRNA pool, JNK2 siRNA pool and non-targeting siRNA pool (D-001810-10-20, Negative control) were obtained from Dharmacon and resuspended in RNAase free water at 100 μM. Human JNK1 siRNA pool target sequences were 5'-GCCCAGUAAUAUAGUAGUA-3', 5'-GGCAUGGGCUACAAGGAAA-3', 5'-GAAUAGUAUGCGCAGCUUA-3' and 5'-GAUGACGCCUUAUGUAGUG-3'. Human JNK2 siRNA pool target sequences were 5'-UCGUGAACUUGUCCUCUUA-3', 5'-AGCCAACUGUGAGGAAUUA-3', 5'-GGCUGUCGAUGAUAGGUUA-3' and 5'-GAUUGUUUGUGCUGCAUUU-3'. Cells were seeded on coverslips at 5 × 10^4 ^cells per cm^2 ^in Opti-MeM to give 40% confluency on treatment day. Cells were transfected with pooled JNK1 and JNK2 siRNA or Negative control siRNA in Lipfectamine 2000 for 5 hr at room temperature (0.5 μg RNA per well). Media was then replaced with normal SY5Y growth medium overnight before treatment with paraquat (1 mM) overnight. Cells were then collected for Western blot for JNK or fixed for immunofluoresence of TDP-43 and HuR.

### Western blot analysis of protein expression and phosphorylation

Cell lysates prepared in Phosphosafe Extraction Buffer at equal protein concentration were mixed with electrophoresis SDS sample buffer and separated on 12% SDS-PAGE Tris-Glycine gels. Proteins were transferred to PVDF membranes and blocked with 4% skim milk solution in PBST before immunoblotting for total or phospho-specific proteins. For detection of total TDP-43, membranes were probed with polyclonal antisera (1:1500) against TDP-43. Secondary antiserum was rabbit-HRP at 1:5,000 dilution. For detection of total and phospho-forms of JNK, ERK and p38, membranes were probed with anti-JNK, anti-ERK or anti-p38 (each at 1:5000) and antisera to phospho-forms of each protein (each at 1:5000). Blots were developed using GE Healthcare ECL Advance Chemiluminescence and imaged on a Fujifilm LAS3000 imager (Berthold, Bundoora, Australia). Expression of GAPDH or actin was determined using antisera at 1:5000 and 1:3000 respectively for protein loading controls where necessary.

### Immunofluorescence analysis

SH-SY5Y cells were grown on 12 mm diameter coverslips and treated with stresses as indicated. Cells were fixed with 4% w/v paraformaldehyde in PBS for 30 min and permeabilized with 90% chilled methanol for 5 min. After blocking for 1 hr with 10% normal goat serum, cells were incubated with primary antibody for total TDP-43 (1:1500), ubiquitin (1:150), HuR (1:50), hnRNP A1 (1:200) or hnRNP K (1:200) for 2 hr at room temperature or overnight at 4°C. This was followed by labeling with secondary AlexaFluor or FITC goat anti-mouse or anti-rabbit antisera at 1:500 for 2 hr at room temperature or overnight at 4°C. After washing, the coverslips were incubated with DAPI at 0.5 μg/ml for 5 min and analyzed using a Leica inverted microscope with Zeiss Axiocam digital camera. Images shown are representative of multiple fields and triplicate coverslips per experiment. TDP-43 and HuR-positive stress granules (SGs) were counted in cultures where indicated. A minimum of 500 cells was counted across multiple fields of view (and multiple coverslips) for each treatment. The number of TDP-43 and HuR-positive SGs were counted in these cells. The total number of cells was divided by the total number of SGs to provide a measure of mean SGs per cell. SGs were not observed in untreated cells.

### Preparation of TDP-43 plasmids

Plasmid DNA corresponding to GFP-tagged full-length wild-type (WT) TDP-43 (pEGFP-TDP WT), C-terminal fragments of TDP-43, (pEGFP-TDP 162-414 and pEGFP-TDP 219-414) or empty expression vector pEGFP-C1 were prepared as described by Nonaka et al. [15]. Briefly, plasmid DNA was used to transform MAX Efficiency^® ^DH5α™ Competent Cells (Invitrogen, Mount Waverley, Victoria, Australia) as described by the manufacturer. Transformants were grown and colonies were picked based on kanamycin-resistance and grown in liquid culture for subsequent plasmid purification. DNA was purified using the Wizard^® ^*Plus *Midiprep DNA Purification System (Promega Corporation) as per manufacturer's instructions. DNA was quantified and TDP-43 inserts were identified positively by digestion with *BamHI *and *XhoI*.

### Transfection and expression of plasmids

SH-SY5Y cells were seeded at 2 × 10^5 ^cells per well in 24 well-plates on coverslips. Cells were transfected 24 hr after seeding with the pEGFP-C1 empty vector, pEGFP-TDP WT, pEGFP-TDP 162-414 and pEGFP-TDP 219-414 using Attractene (Qiagen) according to manufacturer's instructions. After 48 hr incubation, cells were fixed with 4% w/v paraformaldehyde in PBS for 30 min. and permeabilized with 90% chilled methanol for 5 min. After washing, the coverslips were incubated with DAPI at 0.5 μg/ml for 5 min and analyzed using a Leica inverted microscope with Zeiss Axiocam digital camera. Expression of TDP-43 was determined by the EGFP-tagged construct. Efficiency of transfection with pEGFP-C1 vector was approximately 20-25%.

### Statistical analysis

All data described in graphical representations are mean ± standard error of the mean (SEM) unless stated from a minimum of three experiments. Results were analysed using a two-tailed Student's *t-*test.

## Abbreviations

ALS: amyotrophic lateral sclerosis; CTF: C-terminal fragment; ERK: extracellular signal-regulated kinase; FTD: frontotemporal dementia; hnRNP: heterogeneous nuclear ribonucleoprotein; JNK: c-JUN N-terminal kinase; SG: stress granule; SOD: superoxide dismutase; TDP-43: TARDP-binding protein 43.

## Competing interests

The authors declare that they have no competing interests.

## Authors' contributions

JM performed cell culture assays, immunofluorescence studies, immunoblotting and contributed to the preparation of the manuscript. SJP performed cell culture assays, immunofluorescence studies, immunoblotting transfected cells with constructs and contributed to the preparation of the manuscript. LJV prepared TDP-43 CTF constructs. KAP prepared, treated and collected cell cultures for analysis. JRL participated in the study design and coordination, contributed to experimental data collection and helped to draft the manuscript. AC performed cell culture assays, immunofluorescence studies, immunoblotting and contributed to the preparation of the manuscript, Q-XL participated in the study design and coordination, contributed to experimental data collection and helped to draft the manuscript. CLM helped to draft the manuscript. TN generated TDP-43 constructs. MH generated TDP-43 constructs. KMK participated in the study design and coordination, contributed to experimental data collection and helped to draft the manuscript. DCHN and MAB developed molecular tools for JNK analysis and contributed to the preparation of the manuscript. PJC participated in the study design and coordination, contributed to experimental data collection and helped to draft the manuscript. ARW conceived the study, participated in the study design and coordination, and helped to draft the manuscript. All authors read and approved the final manuscript.

## Supplementary Material

Additional file 1 **SH-SY5Y cell viability after exposure to nitrosative or oxidative stress inducers**. SH-SY5Y cells were treated with indicated compounds overnight and cell viability was measured with the MTT assay. **A: **Mild neurotoxicity was induced with all compounds tested. Concentrations were SIN-1, 0.1 mM; arginine, 1 mM; Paraquat, 1 mM and 2 mM; 3-NP, 1 mM; MPP+, 2 mM; sodium azide, 5 mM and rotenone, 0.075 mM. **B: **Comparison of neurotoxicity induced by 1 and 2 mM paraquat or 0.05 mM and 0.5 mM arsenite treatment overnight. *p < 0.05, **p < 0.01. n = three experiments. Click here for file

Additional file 2 **Treatment of SH-SY5Y neurons induces SG formation associated with mild toxicity**. Non-differentiated (**A-C**) or differentiated (**D-K**) were treated with 0-2 mM (**A-C**) or 1 mM (**D-K**) paraquat overnight. **A**: Cell viability was determined by MTT assay. **B**: Cell death was determined by LDH assay. **C**: Stress granules (SGs) per cell were determined. *p < 0.05, **p < 0.01. **D-K**: TDP-43 and HuR immunofluorescence was examined in retinoic acid-differentiated neurons after treatment with 1 mM paraquat. Green = TDP-43, Red = HuR, Blue = DAPI. Arrows indicate SGs. Bar = 10 μm. **G **and **K **represent merged images. n = three experiments. Click here for file

Additional file 3 **Paraquat treatment did not induce phosphorylation of TDP-43 in SGs**. Cells were treated overnight with 1 mM paraquat and examined for phosphorylated TDP-43 by immunofluorescence. **A-C: **untreated, **D-F: **paraquat treated. Green = HuR, Red = phospho-TDP-43, blue = DAPI. Bar = 10 μm. **G: **Immunoblot for phospho-TDP-43 (p-TDP-43) in paraquat-treated cultures. Representative images from three separate experiments. Click here for file

Additional file 4 **Treatment of SH-SY5Y cells with different mitochondrial inhibitors did not induce HuR SGs**. Cells were treated with vehicle control (**A-B**), 2 mM MPP+ (**C-D**), 1 mM 3-NP (**E-F**), 0.075 mM rotenone (**G-H**) or 5 mM sodium azide (**I-J**). Cells were analyzed for HuR localization by immunofluorescence. Red = HuR, blue = DAPI. Bar = 10 μm. **K: **Treatment with 1 mM paraquat (PQ) overnight induced phospho-JNK (pJNK) and this was inhibited by co-treatment with 20 μM SP600125. Representative images from three separate experiments. Click here for file

Additional file 5 **Treatment of SH-SY5Y cells with siRNA to JNK inhibits TDP-43 accumulation in SGs**. **A: **Cells were treated with pooled siRNA against JNK1 and JNK2 or with negative control siRNA and examined for JNK expression. siRNA to JNK significantly reduced expression of JNK1 and JNK2. **B-E: **Untreated control cells. **F-I: **cells treated with negative control siRNA reveal TDP-43 and HuR-positive SGs. **J-M: **Cells treated with siRNA to JNK reveal lack of TDP-43 but not HuR-positive SGs. Green = TDP-43, red = HuR, blue - DAPI. Arrows indicate SGs. Bar = 10 μm. Representative images from two-three separate experiments performed in triplicate. Click here for file

Additional file 6 **Treatment of U87MG astroglial and HeLa epithelial cells with paraquat results in TDP-43 SGs**. U87MG (**A-F**) and HeLa (**G-L**) cells were treated overnight with 1 mM paraquat and analyzed for TDP-43 and HuR localization by immunofluorescence. **A-C**: Untreated U87MG cells, **D-F**: paraquat-treated U87MG cells, **G-I**: untreated HeLa cells, **J-L**: paraquat-treated HeLa cells. Green = TDP-43, red = HuR, blue = DAPI. Arrows indicate SGs. Bar = 10 μm. Representative images from three separate experiments performed in duplicate or triplicate. Click here for file
