# Supplementary figures and images for: C-Jun N-terminal kinase controls TDP-43 accumulation in stress granules induced by oxidative stress
Source: Mol Neurodegener. 2011 Aug 8;6:57. doi: 10.1186/1750-1326-6-57 (PMC3162576; doi:10.1186/1750-1326-6-57)

Additional File 1

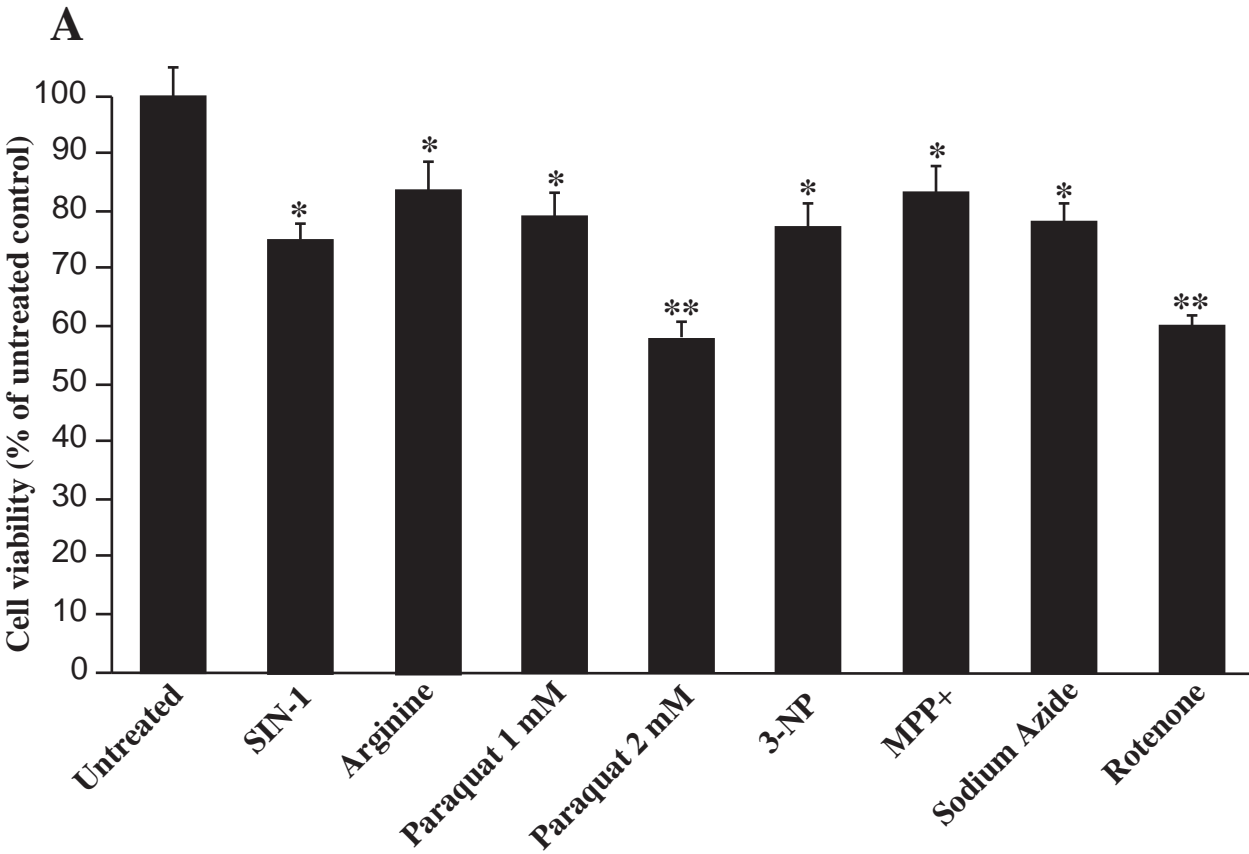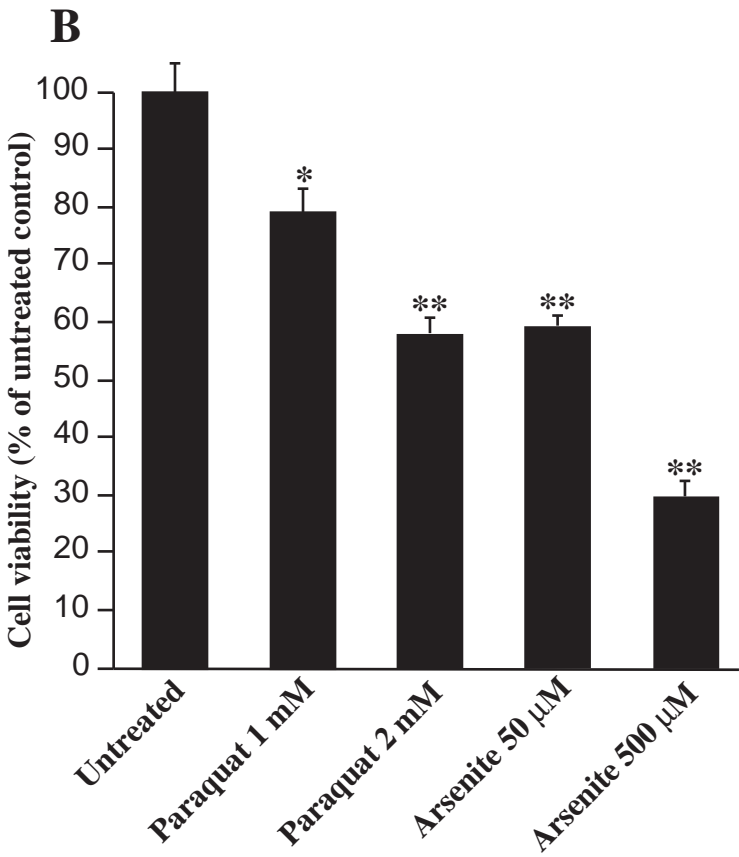

Supplement: Additional file 1 — SH-SY5Y cell viability after exposure to nitrosative or oxidative stress inducers. SH-SY5Y cells were treated with indicated compounds overnight and cell viability was measured with the MTT assay. A: Mild neurotoxicity was induced with all compounds tested. Concentrations were SIN-1, 0.1 mM; arginine, 1 mM; Paraquat, 1 mM and 2 mM; 3-NP, 1 mM; MPP+, 2 mM; sodium azide, 5 mM and rotenone, 0.075 mM. B: Comparison of neurotoxicity induced by 1 and 2 mM paraquat or 0.05 mM and 0.5 mM arsenite treatment overnight. *p < 0.05, **p < 0.01. n = three experiments. [file 1750-1326-6-57-S1.PDF]

Additional File 2

A

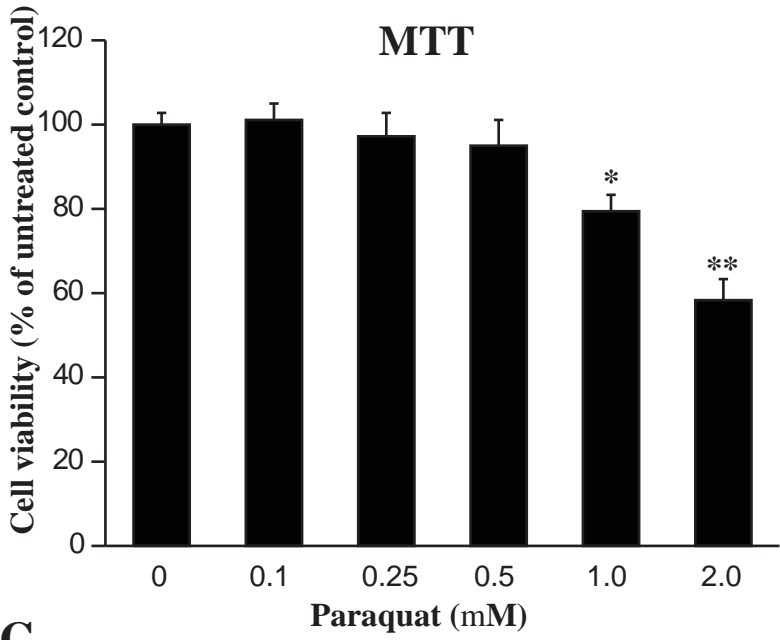

B

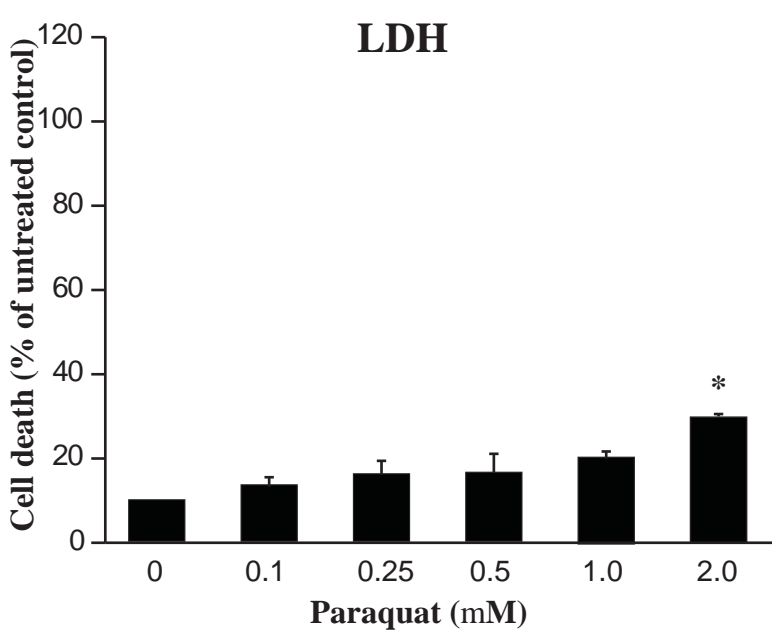

C

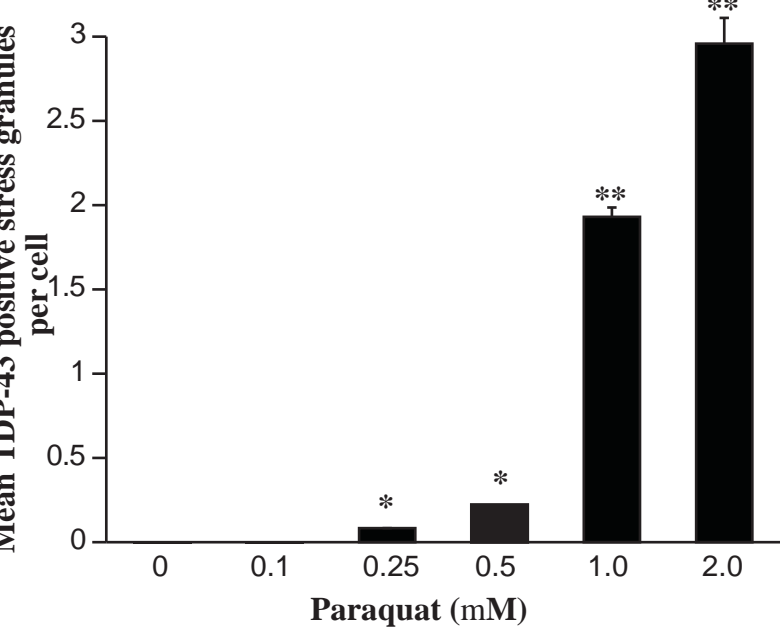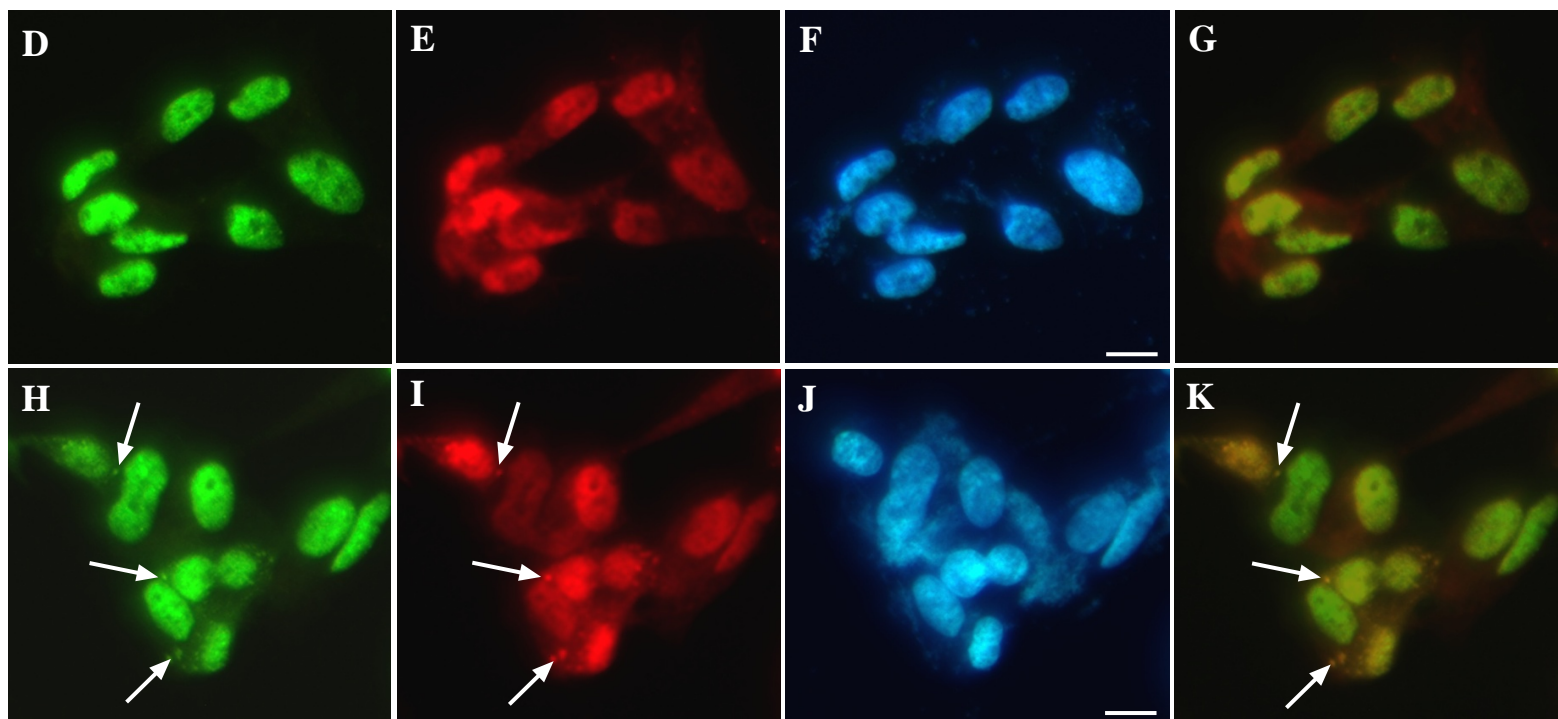

Supplement: Additional file 2 — Treatment of SH-SY5Y neurons induces SG formation associated with mild toxicity. Non-differentiated (A-C) or differentiated (D-K) were treated with 0-2 mM (A-C) or 1 mM (D-K) paraquat overnight. A: Cell viability was determined by MTT assay. B: Cell death was determined by LDH assay. C: Stress granules (SGs) per cell were determined. *p < 0.05, **p < 0.01. D-K: TDP-43 and HuR immunofluorescence was examined in retinoic acid-differentiated neurons after treatment with 1 mM paraquat. Green = TDP-43, Red = HuR, Blue = DAPI. Arrows indicate SGs. Bar = 10 μm. G and K represent merged images. n = three experiments. [file 1750-1326-6-57-S2.PDF]

Additional File 3

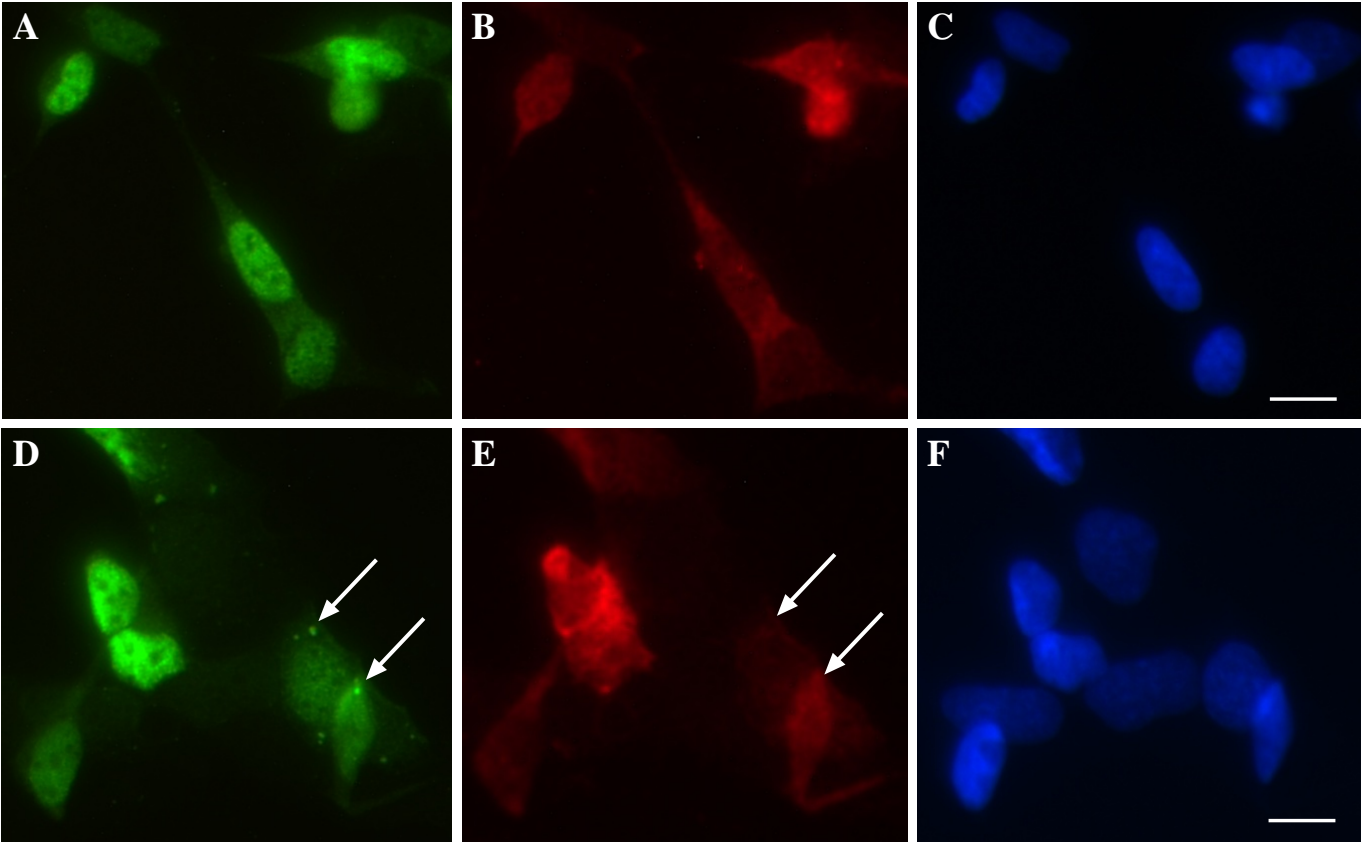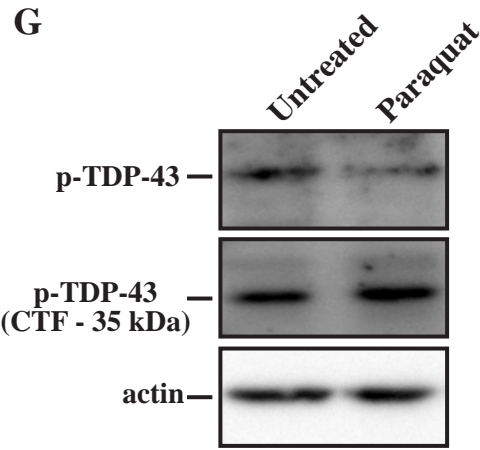

Supplement: Additional file 3 — Paraquat treatment did not induce phosphorylation of TDP-43 in SGs. Cells were treated overnight with 1 mM paraquat and examined for phosphorylated TDP-43 by immunofluorescence. A-C: untreated, D-F: paraquat treated. Green = HuR, Red = phospho-TDP-43, blue = DAPI. Bar = 10 μm. G: Immunoblot for phospho-TDP-43 (p-TDP-43) in paraquat-treated cultures. Representative images from three separate experiments. [file 1750-1326-6-57-S3.PDF]

## Additional File 4

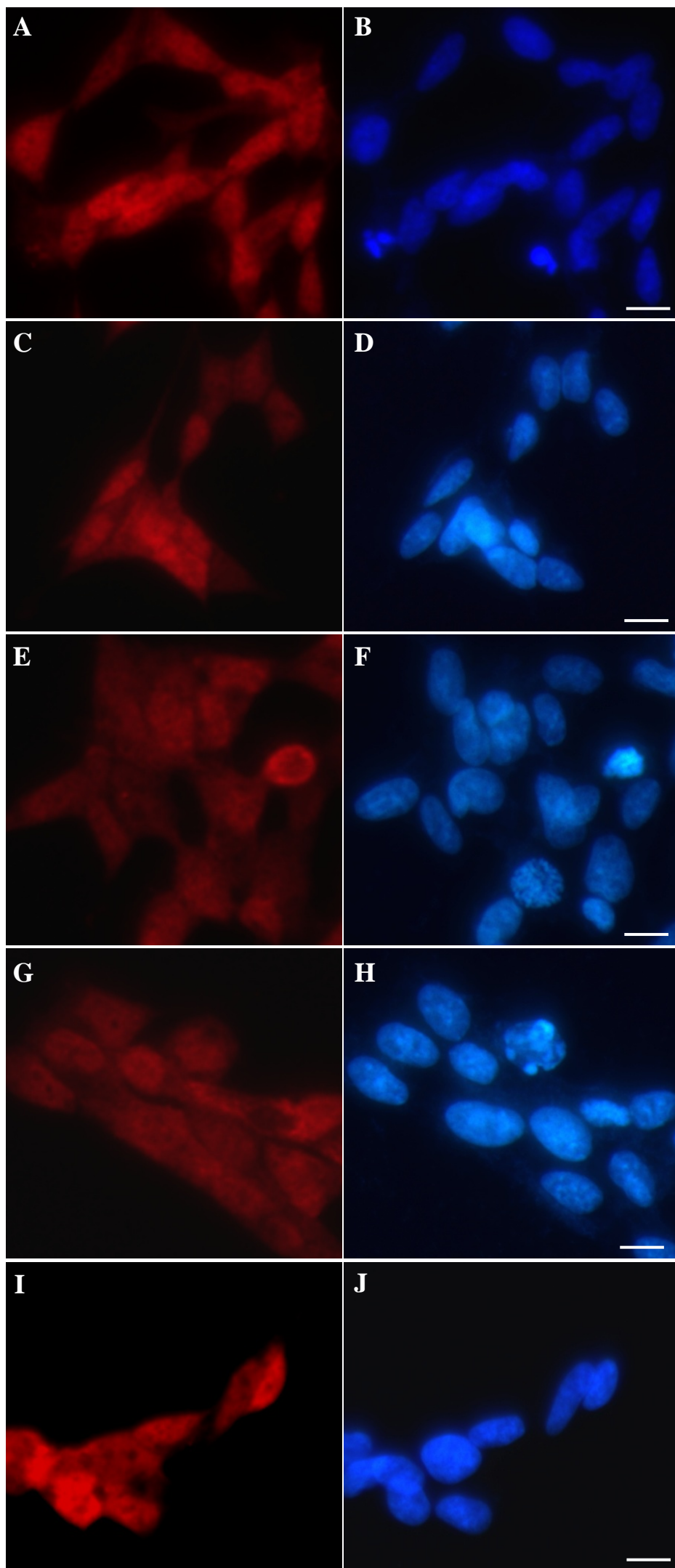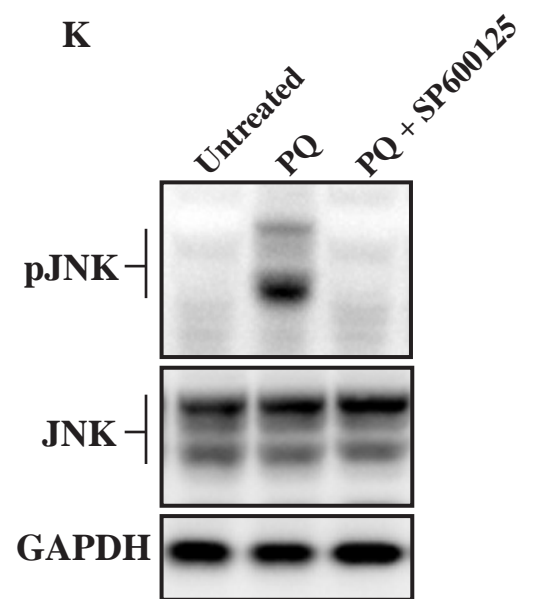

Supplement: Additional file 4 — Treatment of SH-SY5Y cells with different mitochondrial inhibitors did not induce HuR SGs. Cells were treated with vehicle control (A-B), 2 mM MPP+ (C-D), 1 mM 3-NP (E-F), 0.075 mM rotenone (G-H) or 5 mM sodium azide (I-J). Cells were analyzed for HuR localization by immunofluorescence. Red = HuR, blue = DAPI. Bar = 10 μm. K: Treatment with 1 mM paraquat (PQ) overnight induced phospho-JNK (pJNK) and this was inhibited by co-treatment with 20 μM SP600125. Representative images from three separate experiments. [file 1750-1326-6-57-S4.PDF]

Additional File 5

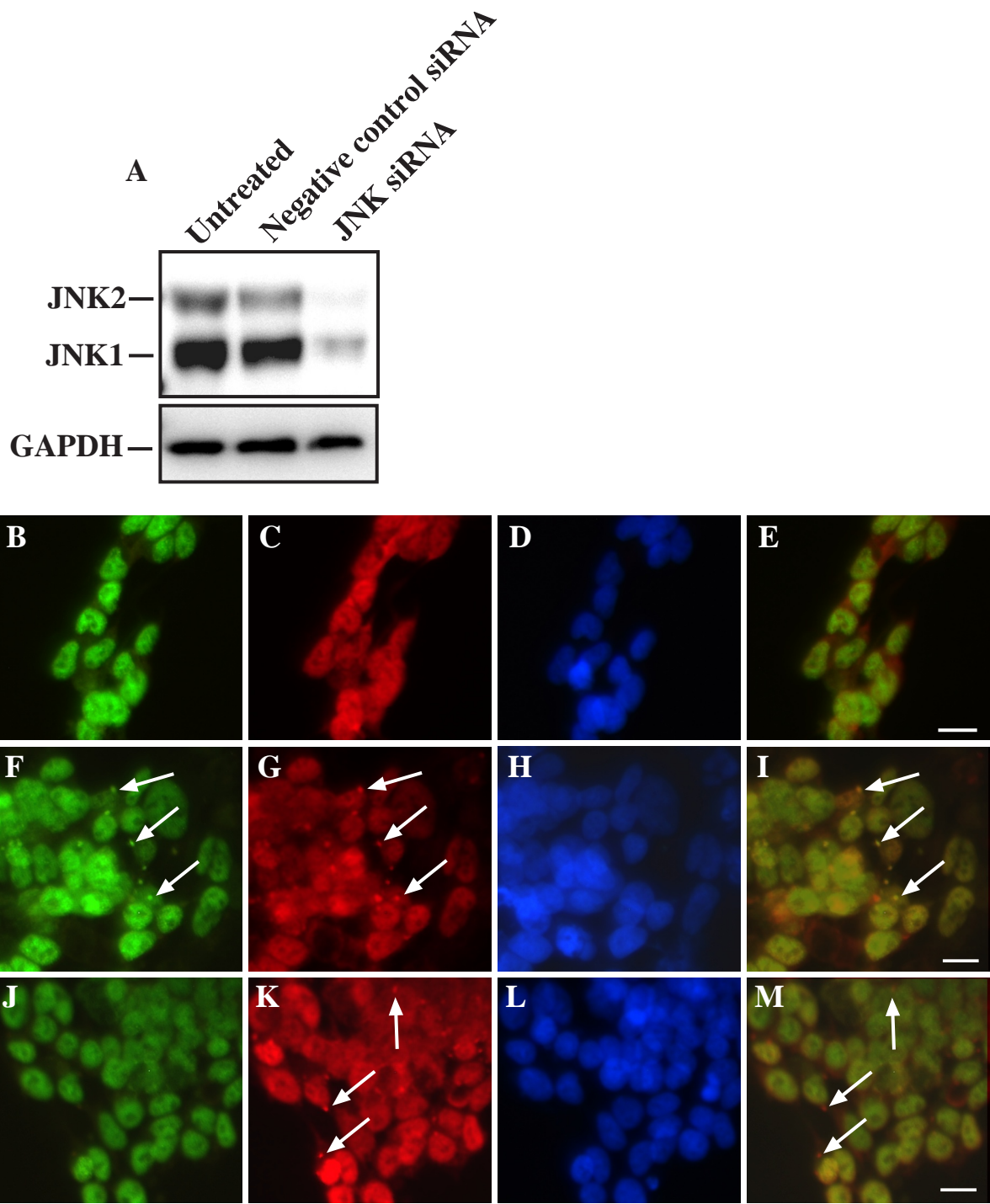

Supplement: Additional file 5 — Treatment of SH-SY5Y cells with siRNA to JNK inhibits TDP-43 accumulation in SGs. A: Cells were treated with pooled siRNA against JNK1 and JNK2 or with negative control siRNA and examined for JNK expression. siRNA to JNK significantly reduced expression of JNK1 and JNK2. B-E: Untreated control cells. F-I: cells treated with negative control siRNA reveal TDP-43 and HuR-positive SGs. J-M: Cells treated with siRNA to JNK reveal lack of TDP-43 but not HuR-positive SGs. Green = TDP-43, red = HuR, blue - DAPI. Arrows indicate SGs. Bar = 10 μm. Representative images from two-three separate experiments performed in triplicate. [file 1750-1326-6-57-S5.PDF]

# Additional File 6

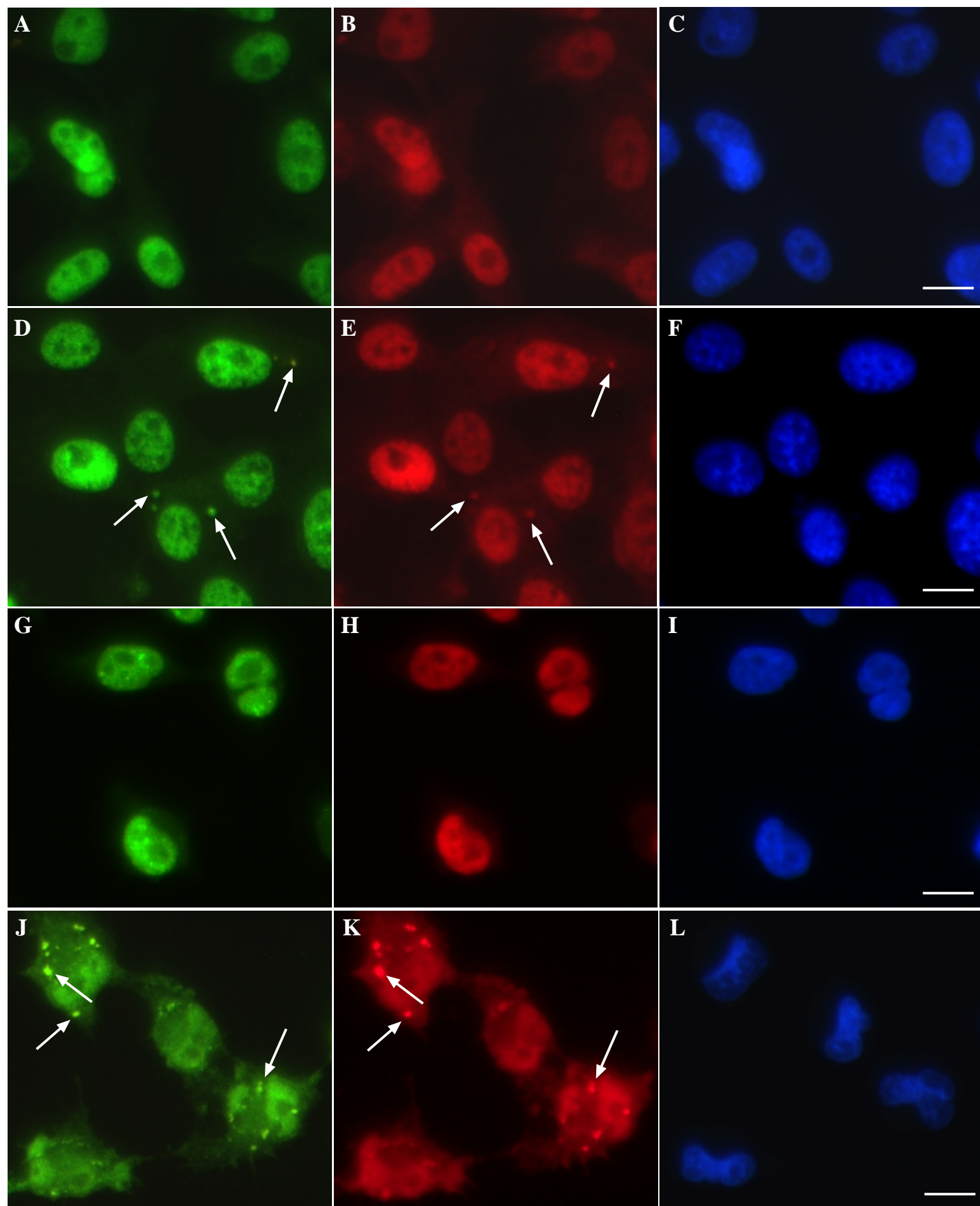

Supplement: Additional file 6 — Treatment of U87MG astroglial and HeLa epithelial cells with paraquat results in TDP-43 SGs. U87MG (A-F) and HeLa (G-L) cells were treated overnight with 1 mM paraquat and analyzed for TDP-43 and HuR localization by immunofluorescence. A-C: Untreated U87MG cells, D-F: paraquat-treated U87MG cells, G-I: untreated HeLa cells, J-L: paraquat-treated HeLa cells. Green = TDP-43, red = HuR, blue = DAPI. Arrows indicate SGs. Bar = 10 μm. Representative images from three separate experiments performed in duplicate or triplicate. [file 1750-1326-6-57-S6.PDF]
